# *PX-RICS*-deficient mice mimic autism spectrum disorder in Jacobsen syndrome through impaired GABA_A_ receptor trafficking

**DOI:** 10.1038/ncomms10861

**Published:** 2016-03-16

**Authors:** Tsutomu Nakamura, Fumiko Arima-Yoshida, Fumika Sakaue, Yukiko Nasu-Nishimura, Yasuko Takeda, Ken Matsuura, Natacha Akshoomoff, Sarah N. Mattson, Paul D. Grossfeld, Toshiya Manabe, Tetsu Akiyama

**Affiliations:** 1 Laboratory of Molecular and Genetic Information, Institute of Molecular and Cellular Biosciences, The University of Tokyo, Bunkyo-ku, Tokyo 113-0032, Japan; 2 Division of Neuronal Network, Department of Basic Medical Sciences, Institute of Medical Science, The University of Tokyo, Minato-ku, Tokyo 108-8639, Japan; 3 Department of Psychiatry, School of Medicine, University of California, San Diego, La Jolla, California 92093, USA; 4 Department of Psychology, San Diego State University, San Diego, California 92120, USA; 5 Department of Pediatrics, School of Medicine, University of California, San Diego, San Diego, California 92123, USA

## Abstract

Jacobsen syndrome (JBS) is a rare congenital disorder caused by a terminal deletion of the long arm of chromosome 11. A subset of patients exhibit social behavioural problems that meet the diagnostic criteria for autism spectrum disorder (ASD); however, the underlying molecular pathogenesis remains poorly understood. *PX-RICS* is located in the chromosomal region commonly deleted in JBS patients with autistic-like behaviour. Here we report that *PX-RICS*-deficient mice exhibit ASD-like social behaviours and ASD-related comorbidities. *PX-RICS*-deficient neurons show reduced surface γ-aminobutyric acid type A receptor (GABA_A_R) levels and impaired GABA_A_R-mediated synaptic transmission. PX-RICS, GABARAP and 14-3-3ζ/θ form an adaptor complex that interconnects GABA_A_R and dynein/dynactin, thereby facilitating GABA_A_R surface expression. ASD-like behavioural abnormalities in *PX-RICS*-deficient mice are ameliorated by enhancing inhibitory synaptic transmission with a GABA_A_R agonist. Our findings demonstrate a critical role of PX-RICS in cognition and suggest a causal link between *PX-RICS* deletion and ASD-like behaviour in JBS patients.

## Abstract (web summary)

The molecular underpinning of autism is unclear. Here the authors show PX-RICS deficient mice exhibit autism-like social behavioural abnormalities and impaired GABA_A_ receptor trafficking, and enhancing inhibitory synaptic transmission with a GABA_A_ receptor agonist ameliorate the behavioural deficits.

## Graphical abstract

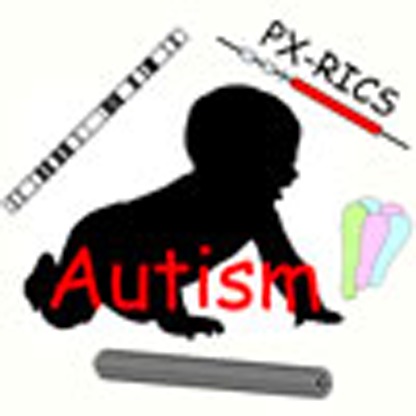

Autism spectrum disorder (ASD) represents a group of aetiologically different neurodevelopmental conditions characterized by a triad of behavioural deficits: impairment in social interaction, difficulty with verbal and non-verbal communication and repetitive behaviour or restricted interests^1^,^2^. ASD is a highly heritable condition, with estimated heritability indices of 80–90% (ref. 3). Several lines of evidence suggest that an imbalance between neuronal excitation and inhibition (E/I) within specific cortical circuits is a shared cellular mechanism for the behavioural and cognitive symptoms in several psychiatric diseases, including ASD^4^,^5^,^6^,^7^. Several known ASD-causative genes encode neuron-associated proteins that are essential for the formation and maintenance of glutamatergic excitatory synapses^8^,^9^. Recent studies have also indicated that impaired presynaptic release of γ-aminobutyric acid (GABA), and the resulting dysfunction of GABA-mediated inhibitory signalling, has been associated with ASD^5^,^10^,^11^,^12^. These findings suggest that a balance between glutamate and GABA signalling is essential for normal cognitive function.

ASD-like behaviour is also known to appear as one of the comorbid symptoms of several developmental syndromes such as fragile-X syndrome^2^,^13^. Studying these syndromes will assist in better understanding the pathogenesis of non-syndromic ASD. Jacobsen syndrome (JBS; MIM 147791), also known as 11q deletion syndrome or partial 11q monosomy syndrome, is a rare congenital disorder caused by a deletion within the distal part of the long arm of chromosome 11 (ref. 14). The incidence of JBS has been estimated to be ∼1 of every 100,000 births^14^. JBS patients commonly suffer from a variety of pre- and post-natal developmental anomalies such as intellectual disability, characteristic facial dysmorphism, heart dysplasia and thrombocytopenia^14^. Prospective genotype/phenotype studies have revealed several candidate genes that may be causative for each symptom of JBS^15^,^16^,^17^. Recently, Akshoomoff *et al*.^18^ have presented evidence that approximately half of JBS patients exhibit behavioural problems that meet the diagnostic criteria for ASD, and identified four annotated genes localized within a tiny (0.24 Mb) interstitial deletion region on 11q24.3 as candidate genes responsible for ASD-like behaviour in JBS. They have concluded that *PX-RICS* is the most promising candidate gene based on its predominant expression in the brain and its critical roles in dendritic spine morphology and axon elongation^18^.

PX-RICS (ARHGAP32 isoform 1), a longer splicing isoform of RICS (ARHGAP32 isoform 2), is a GTPase-activating protein (GAP) for Cdc42, containing multiple domains for protein–protein interactions^19^,^20^. RICS is expressed exclusively in the brain in humans and regulates *N*-methyl-D-aspartate (NMDA) receptor-mediated signalling at the postsynaptic density and axonal elongation at the growth cone^19^,^21^. In contrast, PX-RICS is expressed in relatively wide range of tissues but is predominantly expressed in the brain^20^. PX-RICS has a unique phospholipid-binding domain not shared by RICS: the Phox-homology (PX) domain that mediates its interaction with phosphatidylinositol-4-phosphate (PI4P)^20^. Recently, we have shown that PX-RICS interacts with GABA type A receptor (GABA_A_R)-associated protein (GABARAP) and 14-3-3ζ/θ to facilitate trafficking of the N-cadherin/β-catenin cell adhesion complex in non-neuronal cells^22^,^23^.

In this study, we investigated the molecular mechanisms underlying the autistic-like behaviour of *PX-RICS*-deficient mice. Our results demonstrate an essential role for PX-RICS in normal cognitive function and suggest that PX-RICS deficiency contributes to the behavioural deficits in JBS patients.

## Results

### *PX-RICS*-deficient mice exhibit ASD-like social behaviour

**Figure 1:**
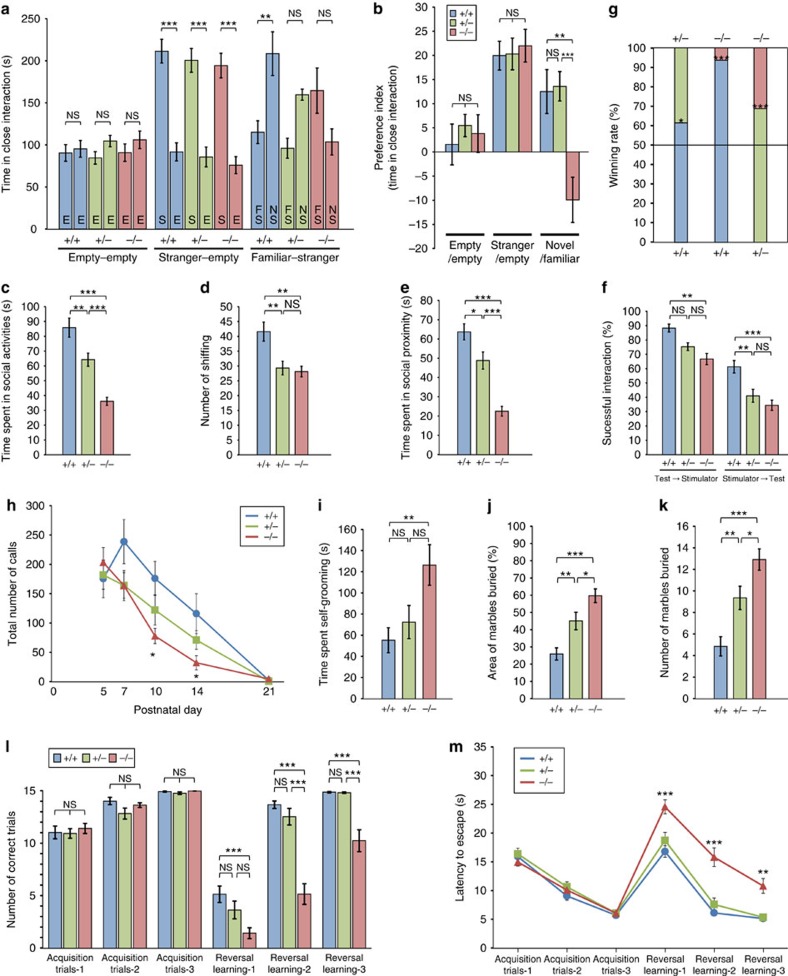
***PX-RICS***^−/−^ **mice exhibit behavioural features similar to the core triad of impairments in ASD.** (**a**,**b**) Three-chamber social interaction test. The time spent in close interaction with each wired cage in each session is shown (**a**). E, empty; FS, familiar stranger; NS, novel stranger; S; stranger. Voluntary sociability and social preference are represented as a preference index, the ratio between time spent near the left and right wired cages in each session (**b**; see Methods). The negative value in *PX-RICS*^−/−^ mice in the familiar–stranger session (novel/familiar) shows their preference for a familiar stranger mouse. (**c**–**f**) Dyadic social interaction test. *PX-RICS*^−/−^ mice spent less time in social activities (**c**–**e**) and showed a markedly lower success rate of social interaction in response to stimulator-initiated approach (**f**). (**g**) Social dominance tube test. *PX-RICS*^−/−^ mice have a markedly lower winning rate. (**h**) The number of USVs of *PX-RICS*^+/+^ mice peaked on PND7 and decreased gradually, whereas that of *PX-RICS*^−/−^ mice were significantly lower, without a peak of calls. (**i**–**k**) *PX-RICS*^−/−^ mice spent more time performing repetitive behaviours such as self-grooming (**i**) and digging (**j**,**k**). (**l**,**m**) Water T-maze test. In the reversal learning session, *PX-RICS*^−/−^ mice showed a lower number of successful (correct arm choice) trials (**l**) and required more time to reach an escape platform (**m**), which markedly contrasts with their normal learning ability in the acquisition session. Data are represented as means±s.e.m. NS, not significant; *P<0.05; **P<0.01; ***P<0.001. One-way analysis of variance (ANOVA) with Tukey's *post hoc* test (**a**–**e**,**h**–**k**), two-way ANOVA with Bonferroni's *post hoc* test (**f**,**l**,**m**) and two-tailed binomial test (**g**).

JBS is a haploinsufficiency disorder in which most cases carry a *de novo* terminal deletion of one chromosome 11q (ref. 14). We thus included *PX-RICS*^+/−^ mice in addition to *PX-RICS*^+/+^ and *PX-RICS*^−/−^ mice in all behavioural assays. We first examined voluntary social interaction using the three-chamber test (Supplementary Fig. 1a)^24^. In the empty–empty (habituation) session, no significant differences were observed between the time spent exploring both wired cages (Fig. 1a,b) and between the time stay in left and right chambers (Supplementary Fig. 1b,c) in all genotypes. When a stranger mouse was placed in one wired cage (stranger–empty session), every genotype had a similar preference for the stranger-containing cage or chamber (Fig. 1a,b and Supplementary Fig. 1b,c), suggesting that *PX-RICS*^−/−^ mice have normal voluntary sociability. In the subsequent familiar–stranger session in which a novel stranger mouse was placed in the previously empty wired cage, *PX-RICS*^+/+^ mice spent more time with the novel stranger mouse (Fig. 1a,b and Supplementary Fig. 1b,c). In contrast, *PX-RICS*^−/−^ mice did not show such a preference for social novelty and instead showed a tendency to spend more time with a familiar stranger mouse (Fig. 1a,b and Supplementary Fig. 1b,c). *PX-RICS*^+/−^ mice exhibited an intermediate phenotype in the familiar–stranger session. No significant differences in exploration and locomotor activity were observed between genotypes in each session (Supplementary Fig. 1d–f).

We next performed a reciprocal social interaction test in which dyadic pairs of test (every genotype) and stimulator (*PX-RICS*^+/+^) mice could freely move and mutually interact in an open arena. We found that *PX-RICS*^−/−^ mice spent less time on social activities (Fig. 1c) such as nose-to-nose sniffing, anogenital sniffing (Fig. 1d) and close huddling (Fig. 1e). In addition, the rate of successful social interactions of *PX-RICS*^−/−^ test mice was significantly lower than that of *PX-RICS*^+/+^ test mice, regardless of whether the approach to the test mouse was initiated by the stimulator or the test mouse itself (Fig. 1f). Interestingly, *PX-RICS*^−/−^ test mice frequently showed apathetic or avoidance responses to approach from the stimulator, resulting in an extremely poor success rate (34.5% in −/− versus 61.4% in +/+) of social interactions. This result suggests that *PX-RICS*^−/−^ mice show normal voluntary social interaction as in the three-chamber test, but show less interest in or avoidance of passive social interaction. *PX-RICS*^+/−^ mice showed intermediate phenotypes.

The results from both social interaction tests demonstrate that PX-RICS deficiency leads to inappropriate or poor social interaction in mice, as is commonly observed in ASD individuals. To further evaluate social interaction, we investigated mice for social dominance and social hierarchies using the tube test, a useful paradigm in predicting impairments in social interaction. In the social dominance tube test, *PX-RICS*^−/−^ rarely won against unfamiliar *PX-RICS*^+/+^ or *PX-RICS*^+/−^ mice (Fig. 1g), suggesting that *PX-RICS*^−/−^ mice are socially subordinate to *PX-RICS*^+/+^ and *PX-RICS*^+/−^ mice, and thus tend to avoid passive social contact with other unfamiliar mice. The test mice displayed no aggressive behaviour; they only pushed their opponent, tried to squeeze themselves into the space between the opponent and the tube wall, or remained still.

Social interaction of nocturnal rodents is primarily driven by olfactory perception^25^. We thus examined responses to olfactory cues using the olfactory habituation/dishabituation test. We found that *PX-RICS*^+/+^ and *PX-RICS*^+/−^ mice show strong habituation/dishabituation responses to all olfactory cues presented (Supplementary Fig. 1g). In contrast, whereas *PX-RICS*^−/−^ mice showed normal responses to non-social odours, they spent much less time interacting with unfamiliar social odours than *PX-RICS*^+/+^ and *PX-RICS*^+/−^ mice. This result suggests that *PX-RICS*^−/−^ mice have decreased interest in unfamiliar social olfactory cues, although their olfactory perception is not impaired.

Mouse pups, when separated from their mother, emit communicative ultrasonic vocalizations (USVs) during the first 2 or 3 postnatal weeks^26^. We thus measured maternal separation-induced USVs to quantify social communication deficits. As shown in Fig. 1h, USVs of *PX-RICS*^+/+^ pups reached a maximum on postnatal day (PND) 7 and then decreased gradually to almost zero by PND21. In contrast, *PX-RICS*^−/−^ pups emitted USVs much less frequently after PND7 and required more time to emit the first call on PND14 (Supplementary Fig. 2a). No significant differences were observed in the mean duration of calls, total calling time, peak frequency and peak amplitude between genotypes (Supplementary Fig. 2b–e). These results suggest atypical postnatal development of mother–pup communication in *PX-RICS*^−/−^ pups.

Repetitive behaviour is the most noticeable symptom in ASD individuals^1^,^2^. We monitored repetitive self-grooming behaviour and found that *PX-RICS*^−/−^ mice spend more than twice as much time on repetitive self-grooming, persisting intermittently for an unusually long (more than 2 min) period (Fig. 1i). The marble-burying test also revealed that *PX-RICS*^−/−^ mice have more than twofold higher activity of repetitive digging behaviour (Fig. 1j,k and Supplementary Fig. 2f). *PX-RICS*^+/−^ mice exhibited intermediate phenotypes in these assays. These results clearly demonstrate increased repetitive behaviour in *PX-RICS*^−/−^ mice.

ASD individuals often exhibit inflexible adherence to routines^1^,^2^. To assess the ability of mice to switch flexibly from a previously established habit to a novel habit, we employed acquisition and reversal learning tasks in a water T-maze^27^,^28^. Learning performance was represented by the number of trials in which mice made the correct arm choice, the number of trials required to achieve five consecutive correct arm choices and the latency to reach an escape platform. In the acquisition session, *PX-RICS*^−/−^ mice showed good learning performance comparable to *PX-RICS*^+/+^ and *PX-RICS*^+/−^ mice (Fig. 1l,m and Supplementary Fig. 2g). In the subsequent reversal learning session, however, *PX-RICS*^−/−^ mice persistently chose the correct arm of the previous acquisition session, resulting in significantly worse learning performance (Fig. 1l,m and Supplementary Fig. 2g). These results suggest that *PX-RICS*^−/−^ mice have a tendency to adhere a previously established habit and less ability to adapt their behaviour to a novel situation.

### *PX-RICS*-deficient mice exhibit ASD-related comorbidities

**Figure 2:**
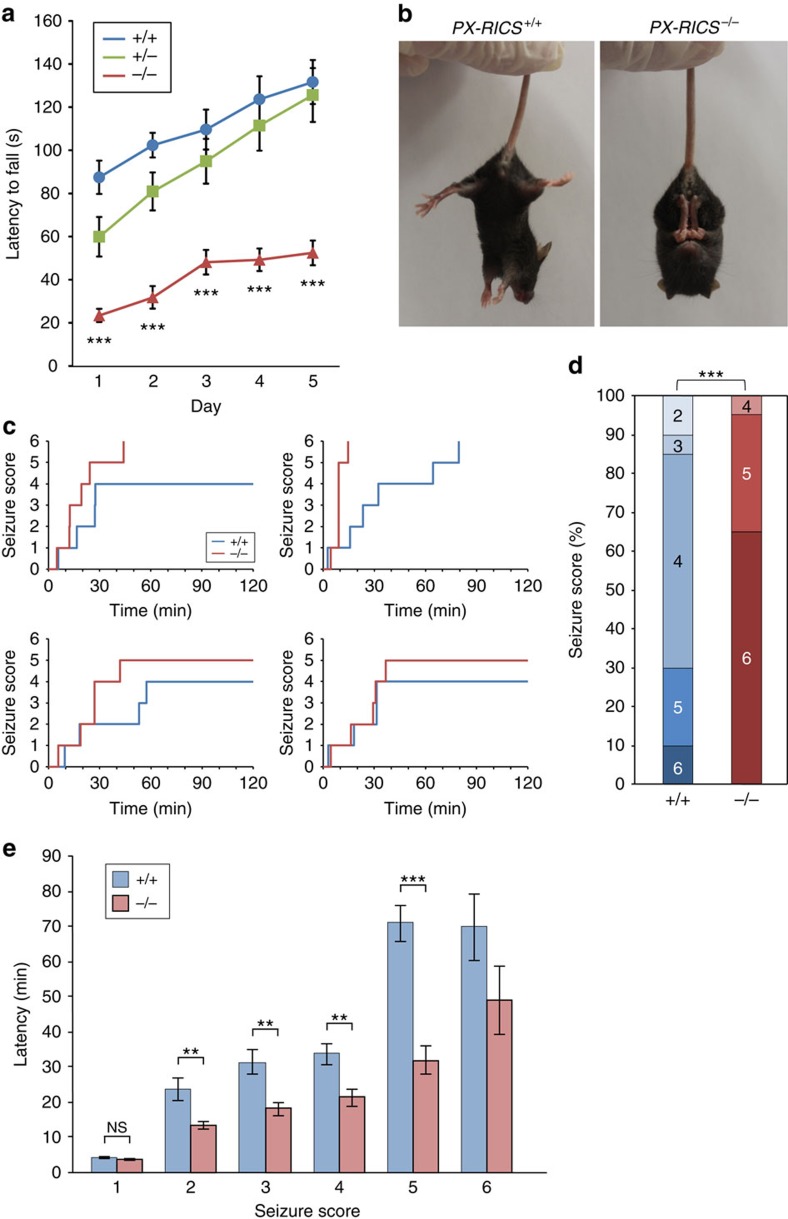
***PX-RICS***^−/−^ **mice display impaired motor coordination and a seizure-prone phenotype.** (**a**) Accelerating rotarod test. *PX-RICS*^−/−^ mice showed poor motor coordination. (**b**) Limb-clasping behaviour in *PX-RICS*^−/−^ mice. When picked up by the tail, *PX-RICS*^+/+^ mice splayed their limbs outwards away from the abdomen. In contrast, *PX-RICS*^−/−^ mice continuously held all four limbs together in a bat-like posture. (**c**–**e**) Increased susceptibility to kainate-induced epileptic seizures in *PX-RICS*^−/−^ mice. (**c**) Representative time-dependent progression of seizure severity in four pairs of *PX-RICS*^+/+^ and *PX-RICS*^−/−^ littermates. (**d**) A breakdown of the maximum seizure severity in *PX-RICS*^+/+^ and *PX-RICS*^−/−^ mice. (**e**) Latency for each seizure stage. Significant differences were not determined in latency to score 6 due to the small number of *PX-RICS*^+/+^ mice that reached the stage. *n*=20 (*PX-RICS*^+/+^) and 20 (*PX-RICS*^−/−^). Data are represented as means±s.e.m. NS, not significant; **P<0.01; ***P<0.001. Two-way analysis of variance with Bonferroni's *post hoc* test (**a**), *χ*^2^-test (**d**) and unpaired two-tailed Student's *t*-test (**e**).

In ASD individuals, the triad of core behavioural problems is commonly accompanied by neurodevelopmental comorbidities, including impaired motor coordination and epilepsy^2^,^13^. We investigated whether these ASD-related phenotypes are present in *PX-RICS*^−/−^ mice. The accelerating rotarod test revealed that *PX-RICS*^−/−^ mice can stay on a rotating rod for significantly less time than *PX-RICS*^+/+^ controls (Fig. 2a), indicating impaired motor coordination. *PX-RICS*^+/−^ mice displayed intermediate motor coordination between *PX-RICS*^+/+^ and *PX-RICS*^−/−^ mice (Fig. 2a).

When suspended by the tail, several mouse models of neuropsychiatric disorders display a limb-clasping phenotype, a dystonic posture characterized by holding the fore- and/or hindlimbs together^29^. This behaviour is a pathological reflex that indicates lesions in the cerebellum, basal ganglia or neocortex^29^. We observed severe limb-clasping behaviour in 68.2% of *PX-RICS*^−/−^ mice (Fig. 2b), which is similar to a mouse model of Rett syndrome, one of the constituent conditions of ASD.

The prevalence of epilepsy is much higher in autistic children (∼30%) than non-autistic individuals (∼1%), which suggests a common molecular basis for cerebral hyperexcitability and impaired cognitive function^2^,^13^. To assess the susceptibility to epilepsy, we scored progressive seizures induced by kainic acid, a specific agonist for the kainate subtype of ionotropic glutamate receptors, with Racine's scale^30^. *PX-RICS*^−/−^ mice were more sensitive to kainic acid than their *PX-RICS*^+/+^ littermates (Fig. 2c). Seizures in the *PX-RICS*^−/−^ mice were more severe (Fig. 2d) and progressed more rapidly to each seizure stage (Fig. 2e).

Taken all behavioural data together, we concluded that PX-RICS deficiency in mice leads to behavioural deficits reflecting the core symptoms of ASD and its related comorbidities. We propose that *PX-RICS* is the gene responsible for ASD-like symptoms in JBS.

### Impaired GABA_A_R surface expression in *PX-RICS* ^−/−^ mice

Results from the behavioural tests prompted us to investigate the cellular function of PX-RICS underlying ASD-like behaviour. We have previously shown that the PX-RICS/GABARAP/14-3-3 complex facilitates dynein-/dynactin-dependent trafficking of the N-cadherin/β-catenin complex in non-neuronal cells^22^,^23^. GABARAP was originally identified as a protein that binds to the γ2 subunit of GABA_A_R and is known to promote GABA_A_R trafficking to the neuronal surface^31^,^32^. We thus hypothesized that the PX-RICS/GABARAP/14-3-3 complex facilitates surface expression of GABA_A_Rs in neurons and that impairment of this trafficking mechanism causes ASD-like behaviour in *PX-RICS*-deficient mice.

**Figure 3:**
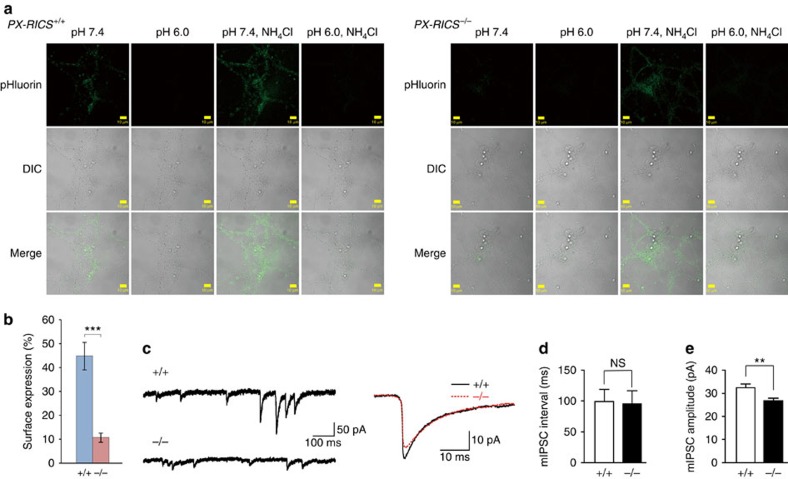
Decreased surface GABA_A_R expression and GABA-mediated inhibitory synaptic transmission in *PX-RICS*^−/−^ neurons. (**a**) Sequential fluorescent (pHluorin) and differential interference contrast (DIC) images of *PX-RICS*^+/+^ (left) and *PX-RICS*^−/−^ (right) live cortical neurons expressing γ2^EP^. The settings of the microscope, lasers and detectors were kept unchanged during image acquisition. Scale bars, 10 μm. (**b**) Decreased surface expression of γ2 in *PX-RICS*^−/−^ neurons. Surface expression was calculated as follows: surface expression (%)=100 × (*I*_7.4_−*I*_6.0_)/(*I*_7.4N_−I_6.0N_), where I_7.4_, I_6.0_, I_7.4N_ and I_6.0N_ represent the fluorescent intensities in the imaging buffer at pH 7.4, pH 6.0, pH 7.4 containing NH_4_Cl and pH 6.0 containing NH_4_Cl, respectively. *n*=20 (*PX-RICS*^+/+^) and 20 (*PX-RICS*^−/−^) fields. (**c**) Representative traces of mIPSCs (left) and averaged traces of mIPSCs (right) in hippocampal CA1 pyramidal cells from *PX-RICS*^+/+^ and *PX-RICS*^−/−^ mice. In total, 221 and 330 consecutive events with no overlap with other events were averaged for *PX-RICS*^+/+^ and *PX-RICS*^−/−^ neurons, respectively. (**d**,**e**) The median intervals (**d**) and amplitudes (**e**) of mIPSCs in *PX-RICS*^+/+^ and *PX-RICS*^−/−^ neurons. *n*=18 (*PX-RICS*^+/+^) and 20 (*PX-RICS*^−/−^) cells (**c**–**e**). Data are represented as means±s.e.m. NS, not significant; **P<0.01; ***P<0.001 (unpaired two-tailed Student's *t*-test).

We first tested the possibility that PX-RICS is involved in GABA_A_R trafficking to the neuronal surface. We utilized γ2^EP^, a γ2 subunit tagged at its N-terminal extracellular domain with ecliptic pHluorin^33^,^34^, a pH-sensitive variant of green fluorescent protein that is non-fluorescent in acidic environments (pH<6)^35^. Thus, γ2^EP^-containing GABA_A_Rs are non-fluorescent during trafficking but become readily visualized when expressed on the neuronal surface. When γ2^EP^ was expressed in *PX-RICS*^+/+^ cortical neurons and observed in pH 7.4 buffer, robust dot-like fluorescent signals could be detected (Fig. 3a). This fluorescence was almost entirely derived from surface-expressed γ2^EP^ because transient exposure to a pH 6.0 buffer quenched the fluorescent signals. Total γ2^EP^ fluorescence was revealed by subsequent exposure to an NH_4_Cl-containing pH 7.4 buffer, which equilibrates the intracellular pH to 7.4. In contrast, in *PX-RICS*^−/−^ cortical neurons, surface fluorescence was markedly reduced, but total fluorescence in pH 7.4 NH_4_Cl-containing buffer was similar to that in *PX-RICS*^+/+^ neurons (Fig. 3a). Quantification of the fluorescence intensities revealed that the amount of surface-expressed γ2^EP^ is markedly decreased in *PX-RICS*^−/−^ neurons (Fig. 3b). This result was confirmed by the cell surface labelling and surface biotinylation assays (Supplementary Fig. 3a,b). Internalization activity for γ2 was similar between the *PX-RICS*^+/+^ and *PX-RICS*^−/−^ cortical neurons, suggesting that the reduction in surface γ2^EP^ in *PX-RICS*^−/−^ neurons is due to impaired trafficking to the plasma membrane, not to enhanced endocytic withdrawal of surface γ2^EP^ (Supplementary Fig. 3e–h). The cell surface labelling and surface biotinylation assays revealed that surface-expressed γ2 is markedly reduced in *PX-RICS*^−/−^ hippocampal neurons and cerebellar granule neurons (CGNs) (Supplementary Fig. 3a,c,d). Taken together, we concluded that surface expression of GABA_A_Rs is impaired in *PX-RICS*^−/−^ neurons.

The efficacy of GABAergic inhibitory transmission is modulated by altering the number of GABA_A_Rs expressed on the neuronal surface^36^,^37^,^38^. We next examined whether reduced surface expression of GABA_A_Rs in *PX-RICS*^−/−^ neurons affects inhibitory synaptic transmission. We measured miniature inhibitory postsynaptic currents (mIPSCs), which reflect postsynaptic responses to GABA released from single synaptic vesicles in the presynaptic terminal, in hippocampal CA1 pyramidal cells using the whole-cell patch–clamp recording technique (Fig. 3c) (ref. 39). Quantitative analysis of mIPSCs revealed no significant difference (*P*=0.89) in the mIPSC frequency (evaluated by the interval between mIPSCs) between *PX-RICS*^+/+^ (99.4±19.4 ms) and *PX-RICS*^−/−^ (95.4±21.4 ms) neurons (Fig. 3d), suggesting that presynaptic neurotransmitter release is similar between *PX-RICS*^+/+^ and *PX-RICS*^−/−^ neurons. In contrast, the mIPSC amplitude of *PX-RICS*^−/−^ neurons (26.8±1.2 pA) was significantly smaller (*P*=0.0075) than that of *PX-RICS*^+/+^ neurons (32.5±1.6 pA; Fig. 3e). These electrophysiological data indicate that postsynaptic responsiveness to inhibitory inputs is attenuated in *PX-RICS*^−/−^ mice, which is in line with the reduced surface expression of GABA_A_Rs.

### PX-RICS forms a trafficking complex for GABA_A_Rs

**Figure 4:**
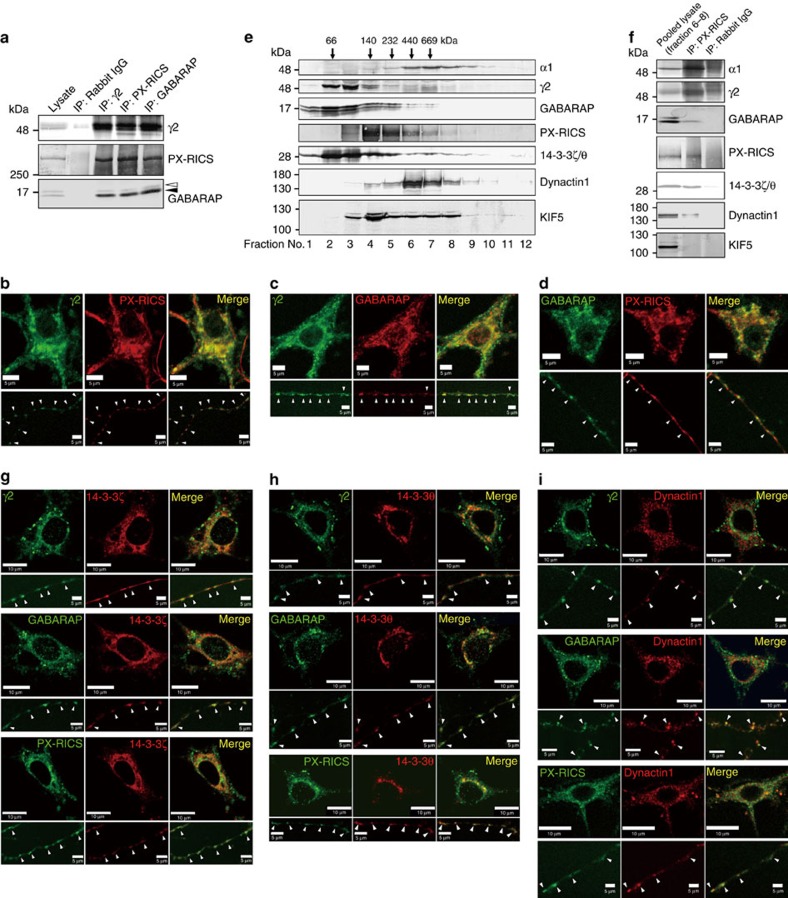
PX-RICS is central to a large cargo-adaptor-motor complex in cortical neurons. (**a**) Co-immunoprecipitation analysis. Note that GABARAP (solid arrowhead) but not GABARAPL1 (open arrowhead) preferentially coprecipitates with γ2 and PX-RICS. (**b**–**d**) Colocalization of PX-RICS, GABARAP and γ2 in the perinuclear region of the soma and in specific compartments of the dendrites (white arrowheads). Scale bars, 5 μm. (**e**) Mouse cortical neurons solubilized with 1% digitonin were fractionated by linear sucrose density gradient (10–40%) ultracentrifugation, and each fraction was subjected to immunoblotting with the indicated antibodies. Arrows at the top indicate the mobilities of the molecular weight markers. (**f**) Fractions 6, 7 and 8 in **e**, the highest-molecular-weight fractions that contain all of the indicated proteins, were pooled and subjected to immunoprecipitation with anti-PX-RICS antibody, followed by immunoblotting with the indicated antibodies. Kinesin superfamily protein 5 (KIF5), a microtubule-dependent molecular motor, was used as a negative control for immunoprecipitation: it is present in fractions 6–8 (**e**), but does not coimmunoprecipitate with PX-RICS in the pooled lysate (**f**). KIF5 is known to interact with GABARAP to facilitate plus-end-directed retrograde transport of GABA_A_Rs (ref. 65). (**g**–**i**) γ2, GABARAP and PX-RICS were colocalized with 14-3-3ζ (**g**), 14-3-3θ (**h**) and dynactin1 (**i**) in the perinuclear region of the soma and specific compartments of the dendrites (white arrowheads). Scale bars, 10 μm (soma); 5 μm (dendrite).

**Figure 5:**
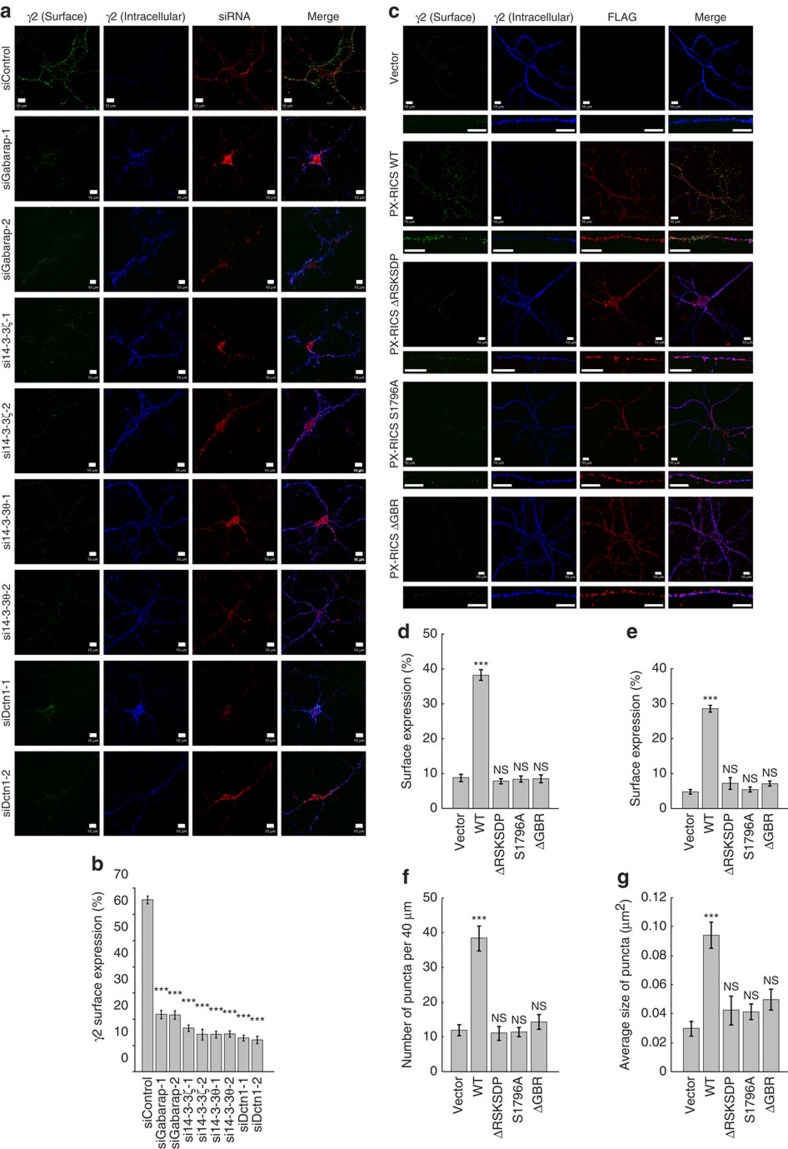
The PX-RICS-dependent trafficking complex is required for surface expression of GABA_A_Rs. (**a**) Mouse cortical neurons (10 DIV) were transfected with the indicated siRNA (10 nM) plus Red Fluorescent Oligo (10 nM). Surface (green) and intracellular (blue) levels of the γ2 subunit in siRNA-introduced neurons (red) were analysed by surface labelling. The settings of the microscope, lasers and detectors were kept constant during image acquisition. Scale bars, 10 μm. (**b**) Quantitative analysis of surface-expressed and intracellular γ2. *n*=10 (images in each siRNA). (**c**) *PX-RICS*^−/−^ cortical neurons (14 DIV) were transfected with plasmids expressing FLAG-tagged wild-type (WT) or mutant PX-RICS as indicated. ΔRSKSDP, a mutant lacking the 14-3-3-binding motif (amino acids 1,793–1,798); S1796A, a mutant in which Ser-1796, the CaMKII phosphorylation site in the 14-3-3 binding motif, is replaced with Ala; ΔGBR, a mutant lacking the GABARAP-binding region (amino acids 562–796). Surface (green) and intracellular (blue) levels of the γ2 subunit in transfected neurons (red) were analysed by surface labelling. The settings of the microscope, lasers and detectors were kept constant during image acquisition. Scale bars, 10 μm. (**d**–**g**) Quantitative analyses of surface-expressed γ2 in entire neurons (**d**) and the representative dendrites (**e**). The number (**f**) and averaged size (**g**) of surface-expressed γ2 puncta within 40-μm portion of the representative dendrites are also quantified using ImageJ. *n*=10 (images in each construct). Data are represented as means±s.e.m. NS, not significant; ***P<0.001 (unpaired two-tailed Student's *t*-test, compared with siControl (**b**) or vector control (**d**–**g**)).

We next analysed whether GABARAP, 14-3-3 and dynein/dynactin participate in PX-RICS-mediated GABA_A_R transport. We found that the γ2 subunit, GABARAP and PX-RICS form a ternary complex in cortical neurons (Fig. 4a) and are colocalized in the perinuclear region of the soma (Fig. 4b–d). Immunofluorescent staining with organelle-specific markers revealed that this region corresponds to the endoplasmic reticulum, endoplasmic reticulum exit sites and endoplasmic reticulum–Golgi intermediate compartments (Supplementary Fig. 4b–l). They were also colocalized in dendritic compartments (Fig. 4b–d) that were immunopositive for endoplasmic reticulum-, endoplasmic reticulum exit site-, endoplasmic reticulum–Golgi intermediate compartment- and *trans*-Golgi network-specific markers (Supplementary Fig. 4b–l), suggesting that PX-RICS and GABARAP are localized in the dendritic satellite secretory outposts^40^,^41^. The colocalization results were confirmed by quantitative analysis of the immunofluorescent signals^42^ (Supplementary Figs 5 and 6). This localization is consistent with their involvement in local delivery of GABA_A_Rs to the dendritic surface. Further ultracentrifugation analysis revealed that 14-3-3ζ/θ and dynactin1, as well as γ2 and GABARAP, coimmunoprecipitated with PX-RICS in high-molecular-weight (>440 kDa) fractions (Fig. 4e,f). We confirmed that 14-3-3ζ/θ and dynactin1 are colocalized with γ2, PX-RICS and GABARAP in the soma and dendrites of cortical neurons (Fig. 4g–i and Supplementary Figs 5 and 6). The introduction of siRNA against any one of them into cortical neurons resulted in a drastic reduction in surface γ2 levels and a simultaneous increase in intracellular γ2 levels (Fig. 5a,b).

We further determined whether the PX-RICS/GABARAP/14-3-3 complex is required for surface expression of GABA_A_Rs. When wild-type PX-RICS was exogenously expressed in *PX-RICS*^−/−^ neurons, surface expression of the γ2 subunit was drastically increased (Fig. 5c–g). In contrast, PX-RICS-ΔRSKSDP and PX-RICS-S1796A, two mutants lacking the binding ability to 14-3-3 (ref. 23), had no apparent effect on surface expression of γ2 (Fig. 5c–g). Similarly, surface γ2 levels remained unchanged in *PX-RICS*^−/−^ neurons expressing PX-RICS-ΔGBR, a mutant defective in GABARAP binding^23^ (Fig. 5c–g).

**Figure 6:**
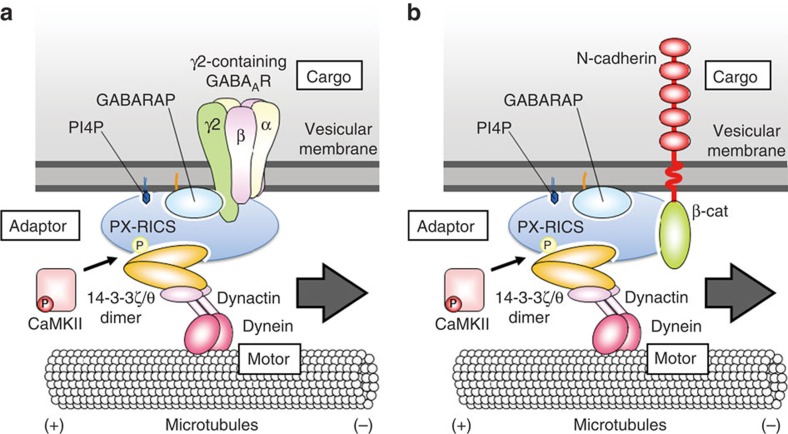
A possible model for a PX-RICS-dependent cargo-motor interconnection. (**a**) The multiple domains mediating protein–protein and protein–phospholipid interactions of PX-RICS enables PX-RICS to play a central role as a scaffold in assembling the constituents of the trafficking complex. Cargo recognition is conducted by GABARAP through its binding to the cytoplasmic region of the γ2 subunit^31^. GABARAP also serves as a binding cue for PX-RICS. Crystal structure analysis has revealed that GABARAP can self-associate in a head-to-tail manner^66^, and this oligomerization activity may underlie its multitasking ability. Phosphorylation of PX-RICS by CaMKII induces the sequential assembly of 14-3-3ζ/θ and dynein/dynactin^23^. By interconnecting the transport-competent γ2-containing GABA_A_R complex with the dynein/dynactin motor complex, the PX-RICS/GABARAP/14-3-3 adaptor complex facilitates GABA_A_R transport from the somatodendritic secretory compartments to the neuronal surface. (**b**) A previously proposed trafficking model for the N-cadherin/β-catenin (β-cat) complex^22^,^23^. Cargo recognition is accomplished by PX-RICS through its binding ability to β-catenin. GABARAP provide a binding cue to PX-RICS to anchor it on the endoplasmic reticulum membrane. See also our previous papers for further details^22^,^23^. Recently, it has become apparent that an increasing number of plasma membrane-bound receptors depend on GABARAP for their cell surface expression^22^,^23^,^32^,^67^,^68^,^69^,^70^. Thus, the PX-RICS-dependent adaptor system may be implicated in the delivery of a wide variety of cell surface proteins.

Taken together, these results suggest that γ2, GABARAP, PX-RICS, 14-3-3ζ/θ and dynactin1 are assembled into a large cargo-adaptor-motor complex that plays a facilitating role in GABA_A_R surface expression. On the basis of a similarity to the trafficking mechanism previously proposed for the N-cadherin/β-catenin complex^22^,^23^, we speculate a possible model for trafficking γ2-containing GABA_A_Rs (Fig. 6).

### A curative effect of clonazepam on autistic-like behaviour

Cell surface labelling revealed that a small amount of the γ2 subunit remains on the surface of *PX-RICS*^−/−^ neurons (Supplementary Fig. 3a). We speculate that this observation may be due to an auxiliary secretory route for γ2-containing GABA_A_Rs that complements or bypasses the PX-RICS-dependent pathway. We reasoned that ASD-like behaviour in *PX-RICS*^−/−^ mice may be treatable by potentiating synaptic inhibition through the GABA_A_Rs remaining on the neuronal surface. Before testing this idea, we first determined an optimum injected dose of clonazepam (CZP), a benzodiazepine agonist for GABA_A_R, to avoid its sedative and anxiolytic effects that could potentially affect our behavioural experiments. Open-field and elevated plus-maze tests revealed that intraperitoneal administration of CZP in a dose up to 0.03 mg kg^−1^ causes no significant sedative or anxiolytic effects in both *PX-RICS*^+/+^ and *PX-RICS*^−/−^ mice (Supplementary Fig. 7a–d).

**Figure 7:**
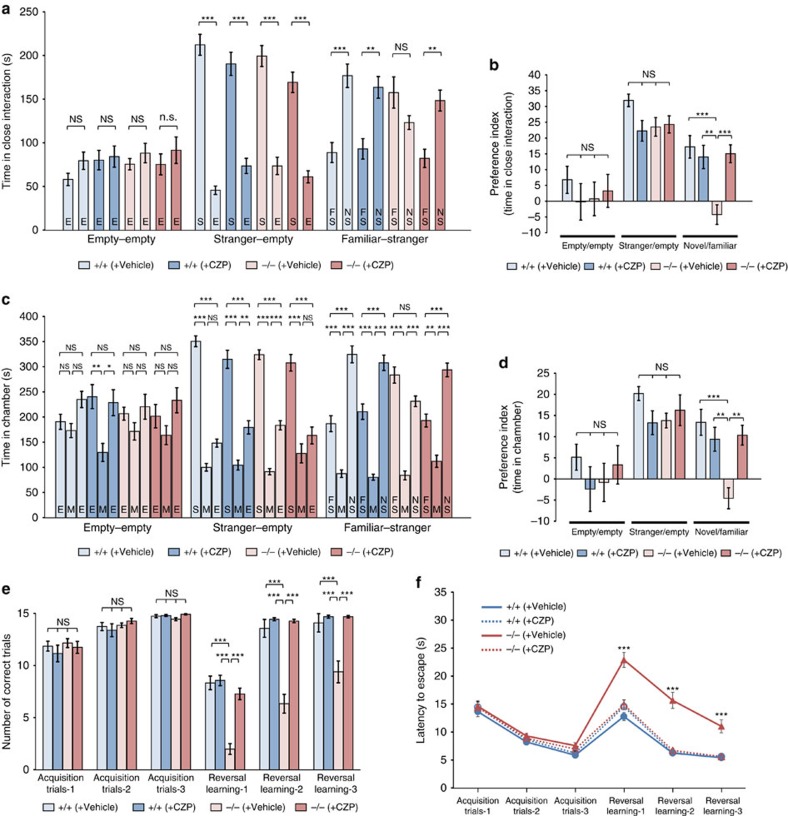
CZP shows an ameliorating effect on impaired social interaction and inflexible behavioural change in *PX-RICS*^−/−^ mice. (**a**–**d**) Three-chamber social interaction test of vehicle- or CZP-treated mice. Time spent near each wired cage (**a**,**b**) and time spent in each chamber (**c**,**d**) are shown. Voluntary sociability and social preference are represented as a preference index (**b**,**d**), as in Fig. 1. In the familiar–stranger session (novel/familiar), CZP-treated *PX-RICS*^−/−^ mice showed a positive preference index comparable to *PX-RICS*^+/+^ mice, indicating that impaired preference for social novelty can be corrected by improving postsynaptic GABA signalling. E, empty; FS, familiar stranger; M, middle chamber; NS, novel stranger; S, stranger. (**e**,**f**) Water T-maze test of vehicle- or CZP-treated mice. CZP-treated *PX-RICS*^−/−^ mice showed reversal learning performance comparable to *PX-RICS*^+/+^ mice in both the number of correct arm choices (**e**) and latency to find an escape platform (**f**). Data are represented as means±s.e.m. NS, not significant; **P<0.01; ***P<0.001. One-way analysis of variance (ANOVA) with Tukey's *post hoc* test (**a**–**d**) and two-way ANOVA with Bonferroni's *post hoc* test (**e**,**f**).

We then conducted behavioural tests of CZP-treated *PX-RICS*^−/−^ mice. In the three-chamber social interaction test, we found that vehicle-treated *PX-RICS*^−/−^ mice show impaired preference for social novelty, whereas preference for social novelty in CZP-treated *PX-RICS*^−/−^ mice is almost completely normalized (Fig. 7a–d). No significant differences were observed in exploration and locomotor activity in each session (Supplementary Fig. 7e–g). Similarly, the water T-maze test revealed that learning performance of CZP-treated *PX-RICS*^−/−^ mice in the reversal learning session is remarkably improved compared with vehicle-treated *PX-RICS*^−/−^ mice (Fig. 7e,f and Supplementary Fig. 7h), indicating that persistence to a previously established habit is significantly alleviated by CZP administration. In each test, intraperitoneal injection of 0.03 mg kg^−1^ CZP had no effect on the social behaviour of *PX-RICS*^+/+^ mice. These results demonstrate that ASD-like behaviour in *PX-RICS*^−/−^ mice is caused by impaired postsynaptic GABA signalling and that GABA_A_R agonists have the potential to treat ASD-like behaviour in JBS patients and possibly non-syndromic ASD individuals.

## Discussion

JBS is a contiguous gene disorder caused by large terminal deletion of chromosome 11q (ref. 14). Simultaneous loss of multiple genes results in a wide variety of congenital defects, including intellectual disability^14^. Recently, Akshoomoff *et al*.^18^ have conducted cognitive and behavioural assessments of JBS patients using standardized diagnostic procedures and shown that more than half of JBS patients meet the criteria for ASD diagnosis. Furthermore, they defined the 0.24-Mb autism critical region in distal 11q and identified four annotated genes: *KCNJ1*, *KCNJ5*, *TP53AIP1* and *RICS/PX-RICS* (Supplementary Fig. 8) (ref. 18). *KCNJ1* encodes the renal outer medullary potassium channel (ROMK), a member of the inward-rectifying K^+^ channel (K_ir_) family that plays important roles in K^+^ secretion into the tubular lumen in the nephron^43^. Mutations of *KCNJ1* cause antenatal Bartter syndrome type 2, a renal tubular disorder characterized by hypokalaemia, metabolic alkalosis and hyperreninemic hyperaldosteronism with normal blood pressure^43^. *KCNJ5* encodes the G-protein-activated inward rectifier K^+^ channel that regulates aldosterone secretion^44^. Mutations of *KCNJ5* are implicated in primary hyperaldosteronism and cardiac long QT syndrome type 13 (ref. 44). *TP53AIP*, p53-regulated apoptosis-inducing protein 1, is a transcriptional target gene for p53 tumour suppressor protein and is specifically expressed in the thymus^45^. The encoded protein is localized in the mitochondrion and mediates p53-dependent apoptosis^46^. Given their reported localizations and functions, it is unlikely that *KCNJ1*, *KCNJ5* and *TP53AIP1* are involved in cognitive function and cause the ASD-like behaviour observed in JBS. In contrast, *PX-RICS* is the only candidate that is expressed predominantly in the brain and known to play functional roles in neurons^20^. The present study has shown that loss-of-function of PX-RICS in mice results in various ASD-like behaviour associated with deficits in PX-RICS-dependent GABA_A_R surface expression (Supplementary Fig. 8). Our findings strongly support the notion that *PX-RICS* is the gene responsible for ASD-like symptoms in JBS patients and also provide evidence that a gene encoding a trafficking factor is implicated in ASD-like behaviour (Supplementary Fig. 8). We therefore propose that JBS-related ASD (and possibly a subset of ASD) is a trafficking disease.

Our findings also demonstrate that PX-RICS-mediated GABA_A_R trafficking is one of the biological systems that support normal cognitive function. Recently, dysfunctional GABA signalling has been increasingly reported to be associated with ASD^5^. GABA-releasing neuron-specific deletion of the gene for methyl-CpG-binding protein 2 (*Mecp2*), a chromatin-associated transcription factor, in mice leads to a variety of Rett syndrome-like behavioural features^10^. Mice lacking the contactin-associated protein-like 2 gene (*Cntnap2*) encoding a neuronal transmembrane protein of the neurexin superfamily display a reduced number of GABA-releasing interneurons and ASD-related phenotypes^11^. Forebrain interneuron-specific deletion of the *Scn1a* gene, which encodes the α subunit of the sodium channel neuronal type I, in mice results in decreased firing activity of GABA-releasing interneurons and ASD-related behaviour^12^. These findings demonstrate that dysfunction of the presynaptic mechanisms for releasing GABA contributes to ASD-like behaviour. In contrast, the present study has shown that dysfunction of the postsynaptic mechanism for trafficking GABA_A_Rs in GABA-receiving neurons is also associated with ASD-like behaviour. Collectively, GABA signalling is essential for normal cognitive function and seriously affects social behaviour if impaired in either GABA-releasing or GABA-receiving neurons.

Another important finding is that the ASD-like behavioural phenotypes of *PX-RICS*-deficient mice can be successfully rescued by treatment with a low dose of CZP, which suggests that compensatory potentiation of postsynaptic GABAergic signalling could be an effective therapeutic strategy for controlling ASD-like behavioural problems^12^,^47^. Despite the increasing number of children diagnosed with ASD and the high prevalence of ASD of ∼1% of the population, currently available pharmacological therapies remain supportive^8^. A recent whole-exome-sequencing study has identified a rare missense single-nucleotide variation in the *PX-RICS* gene in a non-syndromic ASD patient, suggesting that PX-RICS deficiency can be a pathogenic factor for this type of ASD as well^48^. We believe that our *PX-RICS*-deficient mice can be a unique disease model that properly recapitulates the pathogenic process of ASD-like behaviour in JBS patients and a valuable tool for the discovery of permanent cure drugs, as well as for a better understanding of non-syndromic ASD and related neurodevelopmental disorders.

Our previous reports revealed that PX-RICS is a GAP that selectively targets Cdc42 and that PX-RICS deficiency leads to an increase in neuronal Cdc42 activity^19^,^20^,^21^. We also found that the Cdc42GAP activity is required for PX-RICS-mediated transport of the N-cadherin/β-catenin complex^22^. How is Cdc42 activity connected with PX-RICS-mediated GABA_A_R trafficking? In this context, it is notable that collybistin, a brain-specific GDP/GTP exchange factor for Cdc42, is present in the scaffold protein complex for synaptic GABA_A_Rs and regulates gephyrin-dependent GABA_A_R anchoring and clustering^49^. Collybistin binds to phosphatidylinositols (PIs), with a preference for PI3P and PI4P, through its pleckstrin homology domain^50^, as PX-RICS does^20^. Thus, the balance between the activities of the Cdc42 GDP/GTP exchange factor and GAP (that is, collybistin and PX-RICS) might control the synaptic expression and localization of GABA_A_Rs.

In summary, we have found that surface expression of GABA_A_Rs is impaired in PX-RICS^−/−^ neurons. More importantly, stimulation of GABA signalling with a GABA_A_R agonist improved some autistic-like phenotypes of *PX-RICS*^−/−^ mice. Consequently, we conclude that dysfunctional PX-RICS-mediated GABA_A_R trafficking in postsynaptic neurons is a plausible cause for the observed behavioural and molecular deficits. It is possible that alternative binding partners of PX-RICS are also relevant for GABA_A_R-mediated signalling; PX-RICS is known to interact with more than 60 proteins other than GABARAP and 14-3-3 s, many of which have potential roles in important neuronal processes such as neurite extension, regulation of Rho GTPases pathway and transcriptional regulation. Cleary, further investigation is needed to test the involvement of other putative PX-RICS interactors in GABA signalling, as well as other proteins implicated in complementary or compensatory pathways.

## Methods

### Mice

All animal experiments were reviewed and approved by the University of Tokyo Institutional Animal Care and Use Committee, and were conducted according to the University of Tokyo Guidelines for Care and Use of Laboratory Animals. Mice were housed in clean plastic cages (CLEA Japan) lined with paper bedding (Japan SLC) at a constant temperature of 23 °C in a 12-h light/dark cycle (lights off at 21:00), with food and water available *ad libitum*. *PX-RICS*-deficient mice were generated as described elsewhere^21^. Mutant mice were backcrossed to C57BL/6N (CLEA Japan) background to the F10 generation. The mice used in behavioural and electrophysiological studies were generated by breedings of heterozygous mutant males and females in the C57BL/6N background. Young adult male mice (8–10 weeks old) were used in all behavioural tests except for USV analysis and the foot-clasping test, in which both male and female pups of PND5–21 and PND21 were used, respectively. Unless otherwise noted, all tests were conducted with a naive cohort. Six- to eight-week-old male mice were used for electrophysiological analysis. The embryos and newborns for primary neuronal cultures were generated by crossing wild-type C57BL/6N males and females, or homozygous mutant males and females in the C57BL/6N background. Embryonic day (E) 16–18 embryos were used for primary culture of cortical and hippocampal neurons, and PND7 pups for CGNs.

### Behavioural tests

Behavioural assays of mice were designed and performed in accordance with the standard procedures described previously^51^,^52^,^53^,^54^. Detailed procedures are described in the following sections. Full statistical data for the behavioural tests are presented in Supplementary Table 1.

### Three-chamber social interaction test

Mice were tested for voluntary social interaction as previously described^24^. The three-chamber apparatus (O'HARA & CO., LTD.) was a non-transparent acrylic open arena (61 × 40 × 22 cm) with a removal floor placed in a soundproof chamber (74 × 50 × 83 cm). The arena was divided into left, middle and right chambers (20 × 40 × 22 cm) by two semi-transparent partitions with a small rectangular opening (5 × 3 cm) in the bottom centre, through which a test mouse could travel between the three chambers. A quarter cylindrical wired cage (10 cm in radius and 10.5 cm in height) was placed at each back corner of the left and right chambers. A quarter cylindrical weight (10 cm in radius and 10.5 cm in height) was placed on the top of each wired cage to prevent the test mouse from climbing the wired cage and the stranger mouse from escaping. Illumination on the arena floor was kept at 100 lx during the tests.

Male test mice 8–10 weeks old were habituated to the testing room for at least 1 h before beginning the test. For 3 days before testing, age-matched wild-type males unfamiliar to the test mice (stranger mice) were left inside the wired cages for 20 min per day for habituation. A test mouse was first placed in the centre of the middle chamber and left to habituate for 10 min in the absence of stranger mice in the wired cages (empty–empty session). Subsequently, a stranger mouse was introduced into one of the two wired cages. Then, the test mouse was placed in the centre of the middle chamber and allowed to freely explore all three chambers for 10 min (stranger–empty session). Finally, after a novel stranger mouse was placed in another wired cage that had served as the empty cage in the previous sessions, the test mouse was again allowed to freely explore for 10 min (familiar–stranger session). Movement of the test mouse was recorded by a charge-coupled device camera and analysed online with Time CSI video tracking software (O'HARA & CO., LTD). The open arena, partitions, wired cages and weights were cleaned with 70% ethanol and wiped with paper towels before the next trial. Time spent in close interaction near each wired cage and time spent in each chamber were converted into a preference index, which was calculated as 100 × (time with a right empty cage)/(time with a right empty cage+time with a left empty cage)−50 (in the empty–empty session), as 100 × (time with a stranger mouse)/(time with a stranger mouse+time with an empty cage)−50 (in the stranger–empty session) or as 100 × (time with a novel stranger mouse)/(time with a novel stranger mouse+time with a familiar stranger mouse)−50 (in the familiar–stranger session).

### Dyadic social interaction test

Mice were tested for reciprocal social interaction as previously described^55^. Male test mice 8–10 weeks old were habituated to the testing room for at least 1 h before beginning the test. Wild-type males unfamiliar with the test mice were used as stimulators, and their tail tips were painted black with a marker for identification before habituation. An age-matched pair of test and stimulator mice was placed in a non-transparent acrylic open arena (50 × 50 × 40 cm) lined with fresh paper bedding, and their behaviour was video-recorded for 10 min. The number and duration of the following social behaviour were measured by visual observation of the movie: sniffing (nose-to-nose, anogenital and to other areas (neck, flank, tail and so on)), social pursuit (following within one body length as the stimulator mouse moved continuously throughout the cage), social proximity (close huddling within one body length of each other without movement) and total social activities, as well as the latency to the first social contact. We did not observe other social behaviour, such as allo-grooming and social mounting, in our 10-min tests. In addition, we quantified the responses of the test mice (successful interaction or no interaction (no interest or escape)) when approach to the test mouse was initiated by stimulator or test mouse itself. The open arena was cleaned with 70% ethanol and wiped with paper towels before the next test.

### Social dominance tube test

The tube test for social dominance and social hierarchy was performed as described previously^56^,^57^. The apparatus was a transparent plexiglass tube 30 cm in length and 3.5 cm in diameter. Two age-matched (8–10 weeks old) male mice of different genotypes unfamiliar to each other were placed at opposite ends of the tube and simultaneously released in a forward direction towards each other. Almost all mice entered the tube obediently without any particular training. A mouse was judged as a loser when it backed away and put its four paws outside the tube earlier than its opponent within 2 min. A mouse was judged as a winner when it remained inside the tube after the opponent backed out of the tube. To avoid bias, the same pair of mice was subjected to a second match with no interval, switching the releasing side. All matches ended within 2 min in our test. The tube was cleaned with 70% ethanol and wiped with paper towels between matches.

### Olfactory habituation/dishabituation test

The ability of mice to discriminate odorant stimuli was tested as described previously^12^,^28^. We employed water as a control, almond and banana extracts as unfamiliar non-social odours, male mouse urine as a social odour and food pellets as a familiar non-social odour. The almond (KYORITSU FOODS) and banana (NARIZUKA Corporation) extracts were diluted (1:100) with water. The mouse urine was obtained separately from two age-matched wild-type males unfamiliar to the test mice. Food pellet odorant was prepared by soaking a ground food pellet in water. Cotton swabs saturated with these odorants (50 μl) were presented to mice using a test tube clamp so that the tips of the swabs were fixed 2 cm above the centre of the cage lids to prevent the test mice from touching the swabs with their forepaws. Before beginning the test, a male test mouse 8–10 weeks old was habituated to a cotton swab for 30 min in its home cage. Then, the odours were sequentially presented in three consecutive trials per odorant stimulus (2 min per trial) in the following order: water, almond extract, banana extract, mouse urine 1, mouse urine 2 and food pellet. In each trial, the time spent sniffing towards the swab was measured by an observer with a stopwatch.

### Ultrasonic vocalizations

Maternal separation-induced USVs were monitored as described previously^58^. All procedures were performed in a soundproof chamber (O'HARA & CO., LTD.). Both male and female pups were individually removed from their mother at PND5, 7, 10, 14 and 21, and gently placed inside a stainless steel open cylinder (7.5 cm in diameter and 7 cm in height) on a cooling plate set at 24 °C. Recording was started after a 1-min delay because we commonly observed that immediately after being placed inside the cylinder, pups emitted unusually frequent and strong USVs, regardless of their genotype, sex and PND. After a 5-min recording period, pups were promptly returned to their mother. We utilized the UltraSoundGate system (Avisoft Bioacoustics) for recording and SASLab Pro software (Avisoft Bioacoustics) for quantitative analysis. The cylinder was cleaned with 70% ethanol and wiped with paper towels between recordings.

### Repetitive behaviour

Self-grooming behaviour was examined as described previously^59^. A male mouse 8–10 weeks old was placed in a clean empty cage, left for habituation for 10 min and consecutively video-recorded for 10 min. Cumulative time spent in full-body grooming was measured with a stopwatch.

Digging behaviour was evaluated using the marble-burying test as described previously^60^. Twenty black glass marbles (15 mm diameter) were gently placed evenly spaced apart in a 4 × 5 arrangement on 5-cm-thick fresh paper bedding in a clean cage (27.5 × 17 × 13 cm). After taking a still image of marbles from straight above the cage, a male mouse 8–10 weeks old was gently placed in the centre of the cage and left for free exploration for 30 min. Then, the mouse was removed and a still image of the marbles was taken again from straight above the cage. Digging behaviour was quantitatively evaluated by analysing pre- and post-test images with ImageJ software (National Institutes of Health). Strong colour contrast between the black marbles and the white bedding enabled us to easily define the marble area. The marble was judged as ‘buried' if more than 50% of the marble area was covered by the paper bedding. The number and area of marbles buried were evaluated.

### Water T-maze test

Perseverative behaviour was assessed using the water T-maze as previously described^27^,^28^. The apparatus was a T-shaped transparent acrylic open tank 30 cm deep with a long main arm (96 cm in length and 17.5 cm in width) and two perpendicular side arms (36 cm in length and 17.5 cm in width). A white escape platform (6 × 6 cm, 21.5 cm in height) was designed to fit snugly to the end of the side arms to prevent it from wobbling. The apparatus was filled with tap water at 23±1 °C to a depth of 22.5 cm so that the top of the escape platform was positioned 1 cm below the water surface. The water was coloured white with poster paint (Nicker Colours) to make the submerged escape platform invisible.

In a habituation swim trial on day 1, a male mouse 8–10 weeks old was placed on the water surface at the starting end of the main arm facing towards the side arms and then gently released. After 60 s of free swimming without the escape platform, the mouse was gently wiped with a towel, placed in an empty cage on a warming plate (38 °C setting) for 5 min, and then returned to its home cage. On the following 3 days (day 2–4), acquisition swim trials were performed in which the escape platform was submerged at the end of the right arm of the T-maze. Fifteen consecutive swim trials were performed every 3 days. In each trial, the latency to reach the escape platform and the result of arm choice were recorded. The mouse was removed from the platform after a 20-s stay when it reached the escape platform within 35 s. The mouse was guided onto the escape platform and left for 20 s when it could not find the platform within 35 s. In the last 3 days (day 5–7), reversal learning trials were performed as described above, except that the escape platform was placed at the end of the left arm.

### Accelerating rotarod test

Motor coordination was assessed using an accelerating rotarod apparatus (O'HARA & CO., LTD.) as described previously^28^. A male mouse 8–10 weeks old was placed on a rotating shaft of the apparatus when the rotation speed reached 4 r.p.m., and then, the rotation speed was increased to 40 r.p.m. over 5 min. Latency to fall off the rod was measured. Mice were tested for one trial per day for 5 consecutive days. The shaft was cleaned with 70% ethanol and wiped with paper towels between trials.

### Foot-clasping test

To test foot clasping, both male and female mice on PND21 were suspended by the tail for 30 s.

### Seizure susceptibility test

Kainic acid (SIGMA) was dissolved in physiological saline and administered intraperitoneally to 20 pairs of *PX-RICS*^+/+^ and *PX-RICS*^−/−^ littermates (8- to 9-week-old males; 20.4–26.7 g body weight) at a dose of 30 mg per kg body weight in a total volume of 0.20–0.25 ml. The final dose of kainic acid was determined based on the results of pilot experiments. The mice were monitored and video-recorded in a clear cage for 2 h. The video recordings were used to confirm the visual scoring of seizures. Seizure severity was scored according to a modified Racine's scale^30^: 0, no response; 1, behavioural arrest with mouth and facial twitches; 2, head nodding; 3, forelimb clonus; 4, seizures characterized by rearing; 5, seizures characterized by rearing and falling; and 6, death from continuous convulsions.

### CZP treatment

CZP (Wako) was suspended in vehicle solution (0.5% (w/v) methylcellulose 400 (Wako)/phosphate-buffered saline (PBS)) and diluted with the vehicle solution for injection in a volume of 10 ml kg^−1^ at a dose of 0.03–0.5 mg kg^−1^. The diluted CZP suspension or vehicle solution was intraperitoneally administered to a male test mouse 8–10 weeks old 30 min before behavioural testing.

The sedative effect of CZP was evaluated as described previously^12^. The apparatus for the open-field locomotor activity test was a non-transparent acrylic open arena (O'HARA & CO., LTD) without bedding. The CZP-injected mouse was placed in the centre of the arena, and its movement was monitored by a charge-coupled device camera for 10 min. Total distance moved and total immobile time were analysed online with the Time CSI software (O'HARA & CO., LTD). The arena was cleaned with 70% ethanol and wiped with paper towels between trials.

The anxiolytic effect of CZP was evaluated using an elevated plus-maze as described previously^12^. The apparatus (O'HARA & CO., LTD) was a plus-shaped maze fixed at 50 cm in height above the floor comprising two open and two closed arms (25 cm in length and 5 cm in width). The former had small ledges along their edges to prevent a test mouse from losing its footing, whereas the latter were enclosed by semi-transparent acrylic walls (15 cm in height) on three sides. The CZP-injected mouse was placed in the centre of the maze facing towards one of the closed arms and then gently released into the closed arm. Movement of the mice was video-recorded for 10 min. Time spent in the open and the closed arms, and the number of entries into each arm were measured by visual observation of the movies with a stopwatch. The floors and walls of the maze were cleaned with 70% ethanol and wiped with paper towels between trials.

The three-chamber social interaction test and the water T-maze test of the CZP- or vehicle-injected mice were performed as described above.

### Reagents

Rabbit polyclonal antibodies (pAbs) to PX-RICS and GABARAP were generated as described previously^20^,^22^. Briefly, anti-PX-RICS and anti-GABARAP pAbs were generated by immunizing rabbits with recombinant human PX-RICS (amino acids 53–112) and human GABARAP (full length) fused to glutathione *S*-transferase, respectively. The antibodies were purified by affinity chromatography using columns to which the antigens used for immunization had been linked.

### Cell culture and transfection

Cortical and hippocampal neurons were isolated from E16–18 mouse embryos and plated into 6- or 12-well tissue culture plates or 35-mm glass-bottom dishes precoated with 1 mg ml^−1^ poly-L-lysine (SIGMA) as described previously^19^,^21^. Cells were cultured in Neurobasal medium (Invitrogen) supplemented with B-27 supplement (Invitrogen) and 0.5 mM L-glutamine (Invitrogen). A unit of 10 μM cytosine β-D-arabinofuranoside (Ara-C; SIGMA) was added to the medium for the first 3 days. Half of the medium was changed every 3 days. CGNs were isolated from PND7 pups in the same way except that Neurobasal-A medium (Invitrogen) containing 25 mM KCl was used. Transfection of primary cultured cortical neurons was performed at 14 days *in vitro* (DIV) using FuGENE 6 (Roche) for plasmid constructs and at 10 DIV using Lipofectamine RNAiMAX (Invitrogen) for siRNAs. Three hours after transfection, the media were replaced with conditioned media.

### Fluorescent imaging of pHluorin-tagged γ2 in live neurons

pHluorin is a pH-sensitive green fluorescent protein variant that exhibits minimal fluorescence at acidic pH (pH<6.5) and a robust fluorescent signal at neutral pH^35^. The tagged receptors are thus non-fluorescent during trafficking when the N terminus of the γ2 subunit resides in the acidic lumen of transport vesicles but become readily visualized when exposed to the extracellular neutral pH environment at the cell surface^33^,^34^. A mammalian expression construct for the γ2L isoform tagged with ecliptic pHluorin (γ2^EP^) was a kind gift from Dr Moss (Tufts University). Unlike the γ2S isoform, surface expression of γ2L requires the α and β subunits^61^; thus, this method monitors surface expression only of the correctly assembled receptor complex. pcDNA3.1-α1 and pcDNA3.1-β2 were kind gifts from Dr Kang (Vanderbilt University). The GABA_A_R with the subunit combination α1β2γ2 is the most abundant receptor subtype (∼60% of GABA_A_Rs)^62^. Mouse cortical neurons (14 DIV) cultured in 35-mm glass-bottom dishes were transfected with γ2^EP^ (0.5 μg) together with pcDNA3.1-α1 (0.5 μg) and pcDNA3.1-β2 (0.5 μg) using FuGENE 6 according to the manufacturer's instructions. Three hours after transfection, the media were replaced with conditioned media. Forty-eight hours after transfection, fluorescent and differential interference contrast images were obtained with an LSM510META laser scanning confocal microscope (Zeiss). Just before image acquisition, culture media were replaced with imaging media in the following order. IM-7.4 (standard buffer): 10 mM HEPES (pH 7.4), 125 mM NaCl, 5 mM KCl, 1 mM MgCl_2_, 2 mM CaCl_2_ and 10 mM D-glucose; IM-6.0 (low-pH buffer): the same composition as IM-7.4 except that 10 mM HEPES (pH 7.4) was replaced with 10 mM MES (pH 6.0); and IM-7.4N (NH_4_Cl buffer): the same composition as IM-7.4 except that 50 mM NH_4_Cl was added and the concentration of NaCl was reduced to 75 mM; IM-6.0N (low pH, NH_4_Cl buffer): the same composition as IM-7.4N except that 10 mM HEPES (pH 7.4) was replaced with 10 mM MES (pH 6.0). The settings of the microscope, lasers and detectors were kept unchanged during image acquisition.

### Surface labelling

Surface labelling was performed as described^63^. The rabbit pAb to γ2 (Synaptic Systems) used for surface labelling recognizes the N-terminal extracellular region of the subunit. Primary and secondary antibodies used for surface labelling before fixation were diluted in conditioned medium from 14 DIV cortical or hippocampal neurons or from 10 DIV CGNs. The cell images were obtained with an LSM510META laser scanning confocal microscope (Zeiss). The settings of the microscope, lasers and detectors were kept constant during image acquisition.

Supplementary Fig. 3a: *PX-RICS*^+/+^ and *PX-RICS*^−/−^ cortical or hippocampal neurons (14 DIV) or CGNs (10 DIV) were surface-labelled with rabbit pAb to γ2 (1:500; Synaptic Systems) for 30 min at room temperature (RT) and rinsed in PBS. The neurons were incubated with Alexa Fluor 488-labelled anti-rabbit IgG (1:500; Invitrogen) for 30 min at RT, rinsed in PBS and fixed with 2% paraformaldehyde in PBS for 15 min at RT.

Non-permeabilized conditions in Supplementary Fig. 4a: mouse cortical neurons (14 DIV) were surface-labelled with rabbit pAb to γ2 and chicken pAb to MAP2 (1:10,000; Abcam) for 30 min at RT and rinsed in PBS. The neurons were then incubated with Alexa Fluor 488-labelled anti-rabbit IgG and Alexa Fluor 594-labelled anti-chicken IgY (1:500; Invitrogen) for 30 min at RT and rinsed in PBS. The neurons were finally fixed with 2% paraformaldehyde in PBS for 15 min at RT.

Permeabilized conditions in Supplementary Fig. 4a: after fixation with 2% paraformaldehyde in PBS, neurons were permeabilized with 0.2% Triton X-100 in PBS, followed by staining with rabbit pAb to γ2 and chicken pAb to MAP2 for 60 min at RT and then with Alexa Fluor 488-labelled anti-rabbit IgG and Alexa Fluor 594-labelled anti-chicken IgY for 60 min at RT.

Supplementary Fig. 4b–k: mouse cortical neurons (14 DIV) were surface-labelled with rabbit pAb to γ2 for 30 min at RT and rinsed in PBS. After being fixed with 2% paraformaldehyde in PBS for 15 min at RT, the neurons were incubated with a large excess of unconjugated anti-rabbit IgG (50 μg ml^−1^; SIGMA) for 1 h at RT to block the unlabelled anti-γ2 antibody on the surface. The neurons were then permeabilized with 0.2% Triton X-100 in PBS for 5 min and stained with the indicated combinations of the primary antibodies for 60 min at RT and then with Alexa Fluor 488-, Alexa Fluor 594- and Alexa Fluor 647-labelled secondary antibodies for 60 min at RT. Antibodies to organelle-specific markers were as follows: chicken pAb to calreticulin (1:800; Abcam); goat pAb to Sec23 (1:80; Santa Cruz Biotechnology); goat pAb to ERGIC53 (1:50; Santa Cruz Biotechnology); mouse monoclonal antibody (mAb) to GM130 (1:200; Transduction Laboratories); and mouse mAb to syntaxin 6 (1:100; Transduction Laboratories).

Figure 5a: ninety-six hours after siRNA transfection, mouse cortical neurons were surface-labelled with rabbit pAb to γ2 for 30 min at RT and rinsed in PBS. The neurons were incubated with Alexa Fluor 488-labelled anti-rabbit IgG for 30 min at RT and rinsed in PBS. After being fixed with 2% paraformaldehyde in PBS for 15 min at RT, the neurons were incubated with unconjugated anti-rabbit IgG (50 μg ml^−1^; SIGMA) for 1 h at RT to block the unlabelled primary antibody remaining on the neuronal surface, rinsed in PBS and then permeabilized with 0.2% Triton X-100 in PBS for 5 min. The neurons were again incubated with rabbit pAb to γ2 and then Alexa Fluor 647-labelled anti-rabbit IgG.

Figure 5c: *PX-RICS*^−/−^ neurons were transfected with the indicated expression plasmids. The neurons were surface-labelled with rabbit pAb to γ2 for 30 min at RT and rinsed in PBS. The neurons were incubated with Alexa Fluor 488-labelled anti-rabbit IgG for 30 min at RT and rinsed in PBS. After being fixed with 2% paraformaldehyde in PBS for 15 min at RT, the neurons were incubated with unconjugated anti-rabbit IgG for 1 h at RT, rinsed and permeabilized with 0.2% Triton X-100 in PBS for 5 min. The neurons were again double-stained with rabbit pAb to γ2 and mouse mAb to FLAG (M2; 1:500; SIGMA), and then with Alexa Fluor 647-labelled anti-rabbit IgG and Alexa Fluor 594-labelled anti-mouse IgG.

### Surface biotinylation assay

Biotinylation of neuronal surface proteins was performed as described previously^23^. *PX-RICS*^+/+^ and *PX-RICS*^−/−^ cortical or hippocampal neurons (14 DIV) or CGNs (10 DIV) were washed three times with ice-cold PBS and incubated with 0.25 mg ml^−1^ EZ-Link Sulfo-NHS-LC-Biotin (Thermo Scientific) in PBS for 20 min at 4 °C with gentle agitation. After an immediate rinse with ice-cold quenching buffer (50 mM glycine in PBS), the neurons were further washed three times in ice-cold quenching buffer for 5 min each. The cells were lysed in lysis buffer T (10 mM Tris-HCl (pH 6.8), 140 mM NaCl, 1 mM EDTA, 1% Triton X-100, protease inhibitor cocktail and phosphatase inhibitor cocktail) and cleared lysates (0.5 mg of protein) were incubated with 60 μl of 50% slurry of streptavidin–agarose beads (Thermo Scientific) for 2 h at 4 °C. Beads were washed five times in lysis buffer T and then once with PBS. Bound proteins were analysed by immunoblotting with rabbit pAb to γ2 (1:1,000; Synaptic Systems), mouse mAb to transferrin receptor (1:500, Zymed) and mouse mAb to α-tubulin (1:500; Calbiochem). Band intensities were quantified using ImageJ software.

### Internalization assay

The internalization assay was performed as described^63^. *PX-RICS*^+/+^ and *PX-RICS*^−/−^ cortical neurons (14 DIV) were surface-labelled with rabbit pAb to γ2 as described above. After being rinsed in PBS, the neurons were incubated at 37 °C for 1 h in conditioned medium containing 100 μg ml^−1^ leupeptin (SIGMA) to allow internalization of the labelled surface proteins. The neurons were then incubated with Alexa Fluor 594-labelled anti-rabbit IgG for 30 min at RT to visualize the surface-labelled proteins. After fixation, blocking and permeabilization as described above, internalized proteins were visualized with Alexa Fluor 488-labelled anti-rabbit IgG. The settings of the microscope, lasers and detectors were kept unchanged during image acquisition.

### Electrophysiological analysis

To measure IPSCs, hippocampal slices were prepared, and whole-cell patch–clamp recordings were performed as previously described^39^. Briefly, *PX-RICS*^+/+^ and *PX-RICS*^−/−^ mice 6–8 weeks old were anesthetized deeply with halothane and decapitated, and then, the brains were removed. Transverse hippocampal slices (400-μm thick) were cut with a tissue slicer (Vibratome 1500 or Vibratome 3000) in Krebs–Ringer solution (119.0 mM NaCl, 2.5 mM KCl, 1.0 mM NaH_2_PO_4_, 2.5 mM CaCl_2_, 1.3 mM MgSO_4_, 26.2 mM NaHCO_3_ and 11.0 mM glucose) saturated with 95% O_2_ and 5% CO_2_. The slices were incubated for at least 1 h at RT in an interface-type holding chamber filled with Krebs–Ringer solution, and then, a slice was transferred to a submersion-type recording chamber, where the solution was perfused at 1.5–2.0 ml min^−1^ at 25 °C. Whole-cell patch–clamp recordings were made from pyramidal cells in the CA1 region, and the membrane potential was clamped at −70 mV. A recording patch pipette (3–6 MΩ) was filled with the internal solution (140 mM CsCl, 10.0 mM HEPES, 0.2 mM EGTA, 2.0 mM MgATP, 0.3 mM Na_3_GTP, 8.0 mM NaCl and 5.0 mM QX314 (pH 7.2, 295–305 mOsm)). mIPSCs were recorded in the presence of the non-NMDA-receptor antagonist 6-cyano-7-nitroquinoxaline-2,3-dione (10 μM), the NMDA-receptor antagonist D-(−)-2-amino-5-phosphonopentanoic acid (D-AP5: 50 μM) and the voltage-gated sodium channel blocker tetrodotoxin (1 μM; all from Tocris Bioscience). mIPSCs were analysed using the Mini Analysis (6.0.7) software (Synaptosoft). Of all the events detected, only the events that did not overlap with other events were accepted for mIPSC amplitude analysis, whereas all the events were included in the interval analysis. Any significant difference of the median of mIPSC amplitudes and intervals between the genotypes was evaluated with the unpaired two-tailed Student's *t*-test.

### Immunoprecipitation and immunoblotting

Mouse cortical neurons (14 DIV) were lysed in lysis buffer T. The lysates were precleared with protein A-Sepharose (GE Healthcare) for 1 h at 4 °C. Precleared lysates (100–500 μg of protein) were incubated with 5 μg of the indicated antibody for 1 h at 4 °C, and then, the immunocomplexes were adsorbed to protein A-Sepharose for 1 h at 4 °C. After washing extensively with lysis buffer T, the immunoprecipitates were resolved by SDS–PAGE and transferred to a polyvinylidene difluoride membrane (Millipore). The blots were probed with primary antibodies as indicated and visualized with alkaline phosphatase-conjugated secondary antibodies (Promega). Images have been cropped for presentation (Fig. 4 and Supplementary Fig. 3). Full-size images are presented in Supplementary Figs 9 and 10.

### Immunofluorescence

Mouse cortical neurons (14 DIV) were fixed with cold methanol for 20 min at −20 °C and permeabilized with 0.2% Triton X-100 in Tris-buffered saline for 5 min. The neurons were stained with the indicated combinations of primary antibodies for 60 min at RT. Staining patterns were visualized by incubating with the Alexa Fluor 488-, Alexa Fluor 594- or Alexa Fluor 647-conjugated donkey secondary antibodies (1:500; Invitrogen) for 60 min at RT. The cell images were obtained with an LSM510META laser scanning confocal microscope (Zeiss). For immunofluorescence of 14-3-3ζ/θ and dynactin1, the following antibodies were used: rabbit pAb to 14-3-3ζ (1:50; Santa Cruz Biotechnology); rabbit pAb to 14-3-3θ (1:50; Santa Cruz Biotechnology); and mouse mAb to dynactin1 (1:250; Transduction Laboratories). For double immunofluorescent staining of γ2 and early endosome antigen 1 in Supplementary Fig. 3g, mouse mAb to γ2 (1:500; Synaptic Systems) and rabbit pAb to early endosome antigen 1 (1:100; Cell Signaling Technology) were used. Cell nuclei were visualized with TO-PRO-3 iodide (1:10,000; Invitrogen). Quantitative analyses of the colocalization were performed using ImageJ, based on the previously reported method^42^.

### Sucrose density gradient ultracentrifugation

Density gradient ultracentrifugation was performed as described elsewhere^23^. Mouse cortical neurons were washed twice with ice-cold PBS and lysed in 1 ml of lysis buffer D (25 mM HEPES–KOH (pH 6.8), 150 mM NaCl, 2 mM EDTA, 1% digitonin, protease inhibitor cocktail and phosphatase inhibitor cocktail) at 4 °C for 60 min. After centrifugation at 17,000*g* for 30 min, the supernatant (1.5 mg of protein per 0.5 ml) was layered over an 11.5-ml 10–40% (w/v) linear sucrose density gradient containing 25 mM HEPES–KOH (pH 6.8), 150 mM NaCl and 0.4% digitonin. After centrifugation for 15 h at 35,000 r.p.m. in a Beckman SW40 rotor, 12 fractions each containing 1 ml were collected from the top of the tube and subjected to immunoblotting or immunoprecipitation. The antibodies used in immunoblotting were as follows: rabbit pAb to α1 (1:1,000; Synaptic Systems); rabbit pAb to γ2 (1:1,000; Synaptic Systems); rabbit pAb to 14-3-3ζ (1:100; Santa Cruz Biotechnology); mouse mAb to 14-3-3θ (1:5,000; SIGMA); mouse mAb to dynactin1 (1:250; Transduction Laboratories); and mouse mAb to KIF5 (1:200; Millipore). Protein mobility markers (high-molecular-weight native marker kit; GE Healthcare) were applied to a parallel gradient, and their fraction positions were determined by 2–15% native PAGE, followed by Coomassie brilliant blue staining.

### siRNA-mediated gene silencing

Each subunit of cytoplasmic dynein and dynactin exists in a huge variety of distinct isoforms due to multiple genes and complicated neuron-specific alternative splicing^64^. We thus conducted functional knockdown of the dynein/dynactin motor complex by silencing dynactin1 expression because dynactin1 provides the only documented interaction between dynactin and dynein^64^. The sequences for stealth RNAi siRNAs (Invitrogen) were as follows:

siGabarap-1, 5′- AAACAAGGCAUCUUCAGCACGGAGA -3′; siGabarap-2, 5′- AAAGAAGUCUUCUUCAUGGUGUUCC -3′; si14-3-3ζ-1, 5′- UUGAGGGCCAGACCCAGUCUGAUGG -3′; si14-3-3ζ-2, 5′- UUUGCAAGAGAGCAGGCUUUCUCUG -3′; si14-3-3θ-1, 5′- AGAGGUGUCGGUCUUCUGCUCAAUG -3′; si14-3-3θ-2, 5′- AUCAAACGCCUCUUGGUAGGCUCCU -3′; siDctn1-1, 5′- UUAACUCCUUGAUAACUGUCUCUCG -3′; and siDctn1-2, 5′- UGCCCAUGUAGACUGUGUCAUCUUG -3′.

Stealth RNAi negative control medium GC duplex (Invitrogen) is denoted as siControl in the figures. The primary cultured mouse neurons (10 DIV) were transfected with siRNAs (10 nM) plus Red Fluorescent Oligo (10 nM; Invitrogen) using Lipofectamine RNAiMAX according to the manufacturer's protocol. Three hours after transfection, the media were replaced with conditioned media. The silencing effect was evaluated by immunofluorescence 96 h after transfection. Immunofluorescence and surface labelling of siRNA-transfected neurons were performed as described above.

### PX-RICS mutants

PX-RICS mutants were generated by PCR-based mutagenesis^22^,^23^. PX-RICS-ΔRSKSDP, a mutant lacking the 14-3-3-binding motif (amino acids 1,793–1,798); PX-RICS-S1796A, a mutant in which Ser-1796, the CaMKII phosphorylation site in the 14-3-3 binding motif, was replaced with Ala; PX-RICS-ΔGBR, a mutant lacking the GABARAP-binding region (amino acids 562–796). These mutants were cloned in frame into the mammalian expression vector pcDNA3.1(+)-FLAG.

### Statistical analysis

All experiments were performed blind to genotypes and/or treatments. The number of mice used in each test is included in Supplementary Table 1. Sampling and experimental replicates in cell biological and electrophysiological analyses are described in the figure legends. No criteria were applied for inclusion and exclusion of data. No outliers were taken into account and all collected data were subjected to statistical analyses. All data are represented as means±s.e.m. and analysed using one-way analysis of variance with Tukey's *post hoc* test, two-way analysis of variance with Bonferroni's *post hoc* test, binomial test, Student's *t*-test and *χ*^2^-test. All the statistical analyses were performed using Prism 6 software (GraphPad Software, Inc.). Full statistical data for the behavioural tests are presented in Supplementary Table 1.

## Additional information

**How to cite this article:** Nakamura, T. *et al*. *PX-RICS*-deficient mice mimic autism spectrum disorder in Jacobsen syndrome through impaired GABA_A_ receptor trafficking. *Nat. Commun.* 7:10861 doi: 10.1038/ncomms10861 (2016).

## Supplementary Material

Supplementary Information Supplementary Figures 1-10 and Supplementary Table 1
