# Supplementary material for: PX-RICS-deficient mice mimic autism spectrum disorder in Jacobsen syndrome through impaired GABAA receptor trafficking
Source: Nat Commun. 2016 Mar 16;7:10861. doi: 10.1038/ncomms10861 (PMC4799364; doi:10.1038/ncomms10861)
Supplement: Supplementary Information — Supplementary Figures 1-10 and Supplementary Table 1 [file ncomms10861-s1.pdf]

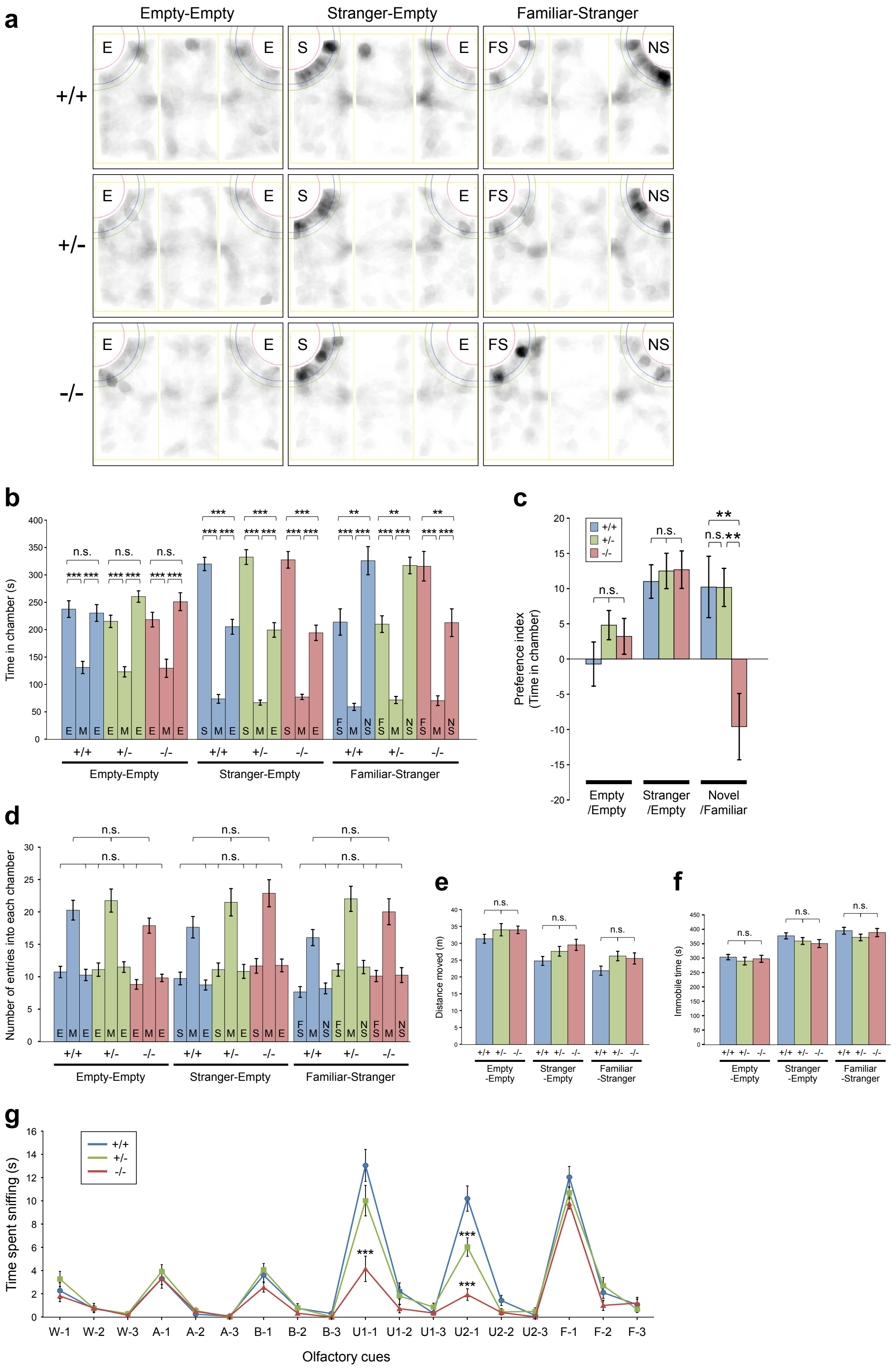

a
Empty-Empty
Stranger-Empty
Familiar-Stranger
+/+
+/-
-/-
E
S
FS
NS
b
Time in chamber (s)
Empty-Empty
Stranger-Empty
Familiar-Stranger
n.s.
c
Preference index (Time in chamber)
Empty /Empty
Stranger /Empty
Novel /Familiar
d
Number of entries into each chamber
e
Distance moved (m)
f
Immobile time (s)
g
Time spent sniffing (s)
W-1 W-2 W-3 A-1 A-2 A-3 B-1 B-2 B-3 U1-1 U1-2 U1-3 U2-1 U2-2 U2-3 F-1 F-2 F-3
Olfactory cues

Supplementary Figure 1 | *PX-RICS*$^{-/-}$ mice show no preference for social novelty and less interest in social olfactory cues

(**a**-**f**) Three-chamber social interaction test. (**a**) Representative heat maps of each session. The density of the black colour reflects the length of time spent at the position. Yellow lines indicate the arena and borders between each chamber. Red circular arcs indicate the borders between wired cages and the chambers. A test mouse was judged to be in close interaction when it stayed inside the blue arcs and away from a wired cage when outside the green arcs. E; empty, S; stranger, FS; familiar stranger, NS; novel stranger. (**b**,**c**) The time spent in each chamber is shown (**b**). M; middle chamber. Voluntary sociability and social preference are presented as a preference index, as in Figure 1 (**c**). (**d**-**f**) No significant difference in exploration (**d**) or locomotor activity (**e**,**f**) between genotypes in each session. (**g**) Olfactory habituation/dishabituation test. W; water, A; almond extract, B; banana extract, U; urine of an unfamiliar male mouse, F; food pellet. *PX-RICS*$^{+/+}$ and *PX-RICS*$^{+/-}$ mice showed much more interest in unfamiliar social odours (U1 and U2) than unfamiliar non-social odours (A and B). In contrast, *PX-RICS*$^{-/-}$ mice showed as much interest in social odour as non-social odour and significantly less interest in social odour than *PX-RICS*$^{+/+}$ mice. Data are represented as means ± s.e.m. n.s.; not significant, $^{**}P<0.01$, $^{***}P<0.001$. One-way ANOVA with Tukey's *post hoc* test (**b**-**f**) and two-way ANOVA with Bonferroni's *post hoc* test (**g**).

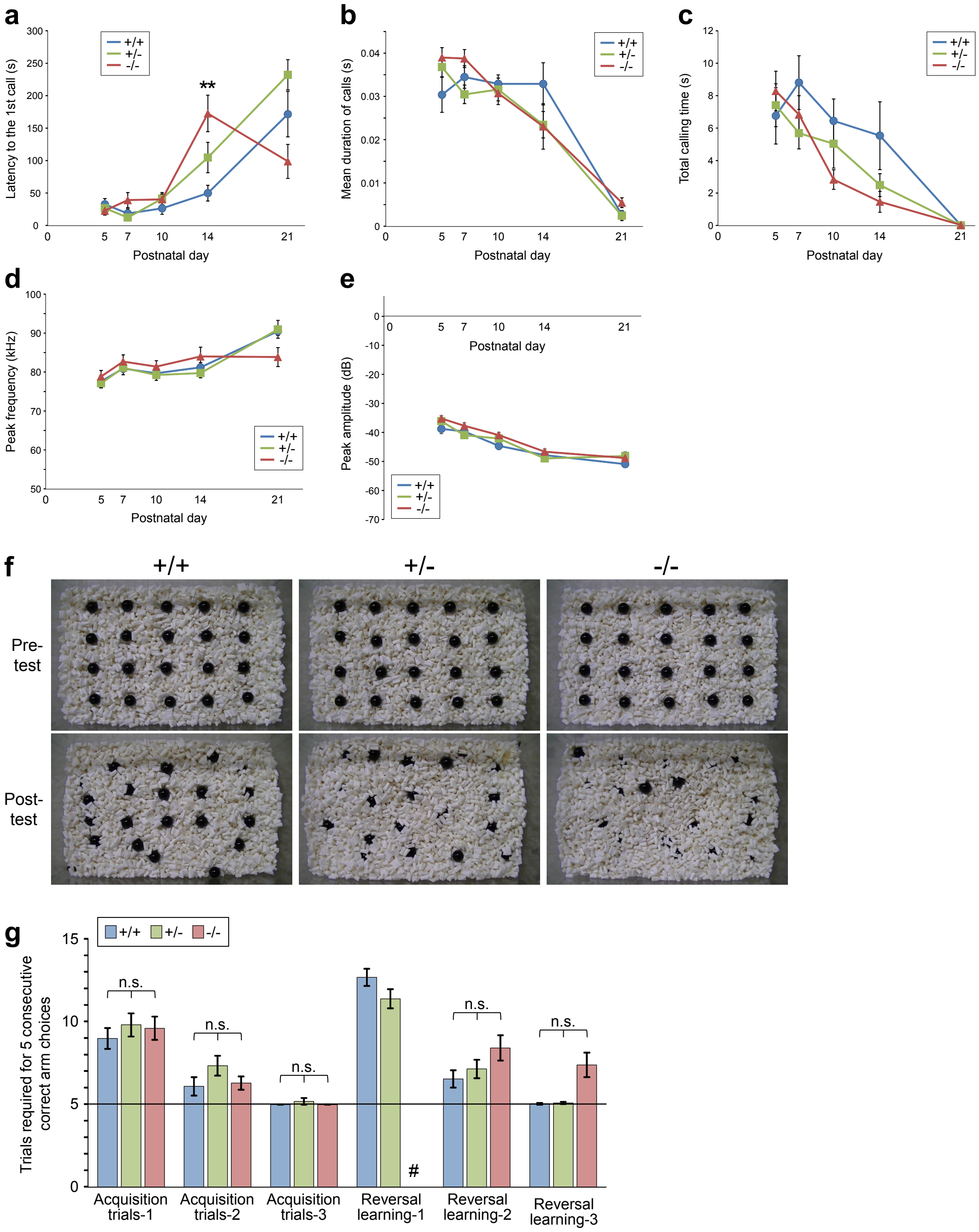

a
Latency to the 1st call (s)
Postnatal day
+/+
+/-
-/-
**
b
Mean duration of calls (s)
Postnatal day
c
Total calling time (s)
Postnatal day
d
Peak frequency (kHz)
Postnatal day
e
Peak amplitude (dB)
Postnatal day
f
+/+
+/-
-/-
Pre-test
Post-test
g
Trials required for 5 consecutive correct arm choices
n.s.
#
Acquisition trials-1
Acquisition trials-2
Acquisition trials-3
Reversal learning-1
Reversal learning-2
Reversal learning-3

Supplementary Figure 2 | *PX-RICS*$^{-/-}$ mice show atypical development of maternal separation-induced USVs, increased repetitive behaviour, and behavioural inflexibility

(**a**-**e**) Maternal separation-induced USVs. The latency to emit the first call (**a**), mean duration of calls (**b**), total calling time (**c**), peak frequency (**d**) and peak amplitude (**e**) are shown. Much more time was required for the first call on PND14 in *PX-RICS*$^{-/-}$ mice. (**f**) Representative pre- and post-test images of marbles in the marble burying test. (**g**) Water T-maze test. The number of trials required for 5 consecutive correct arm choices is shown. As denoted by #, no *PX-RICS*$^{-/-}$ mice could achieve 5 consecutive correct arm choices on day 1 of reversal learning (Reversal learning-1), and the data were removed from the two-way ANOVA. Refer to the statistical analysis data in Supplementary Table 1. Data are represented as means ± s.e.m. n.s.; not significant, $^{**}P<0.01$. One-way ANOVA with Tukey's *post hoc* test (**a**-**e**) and two-way ANOVA with Bonferroni's *post hoc* test (**g**).

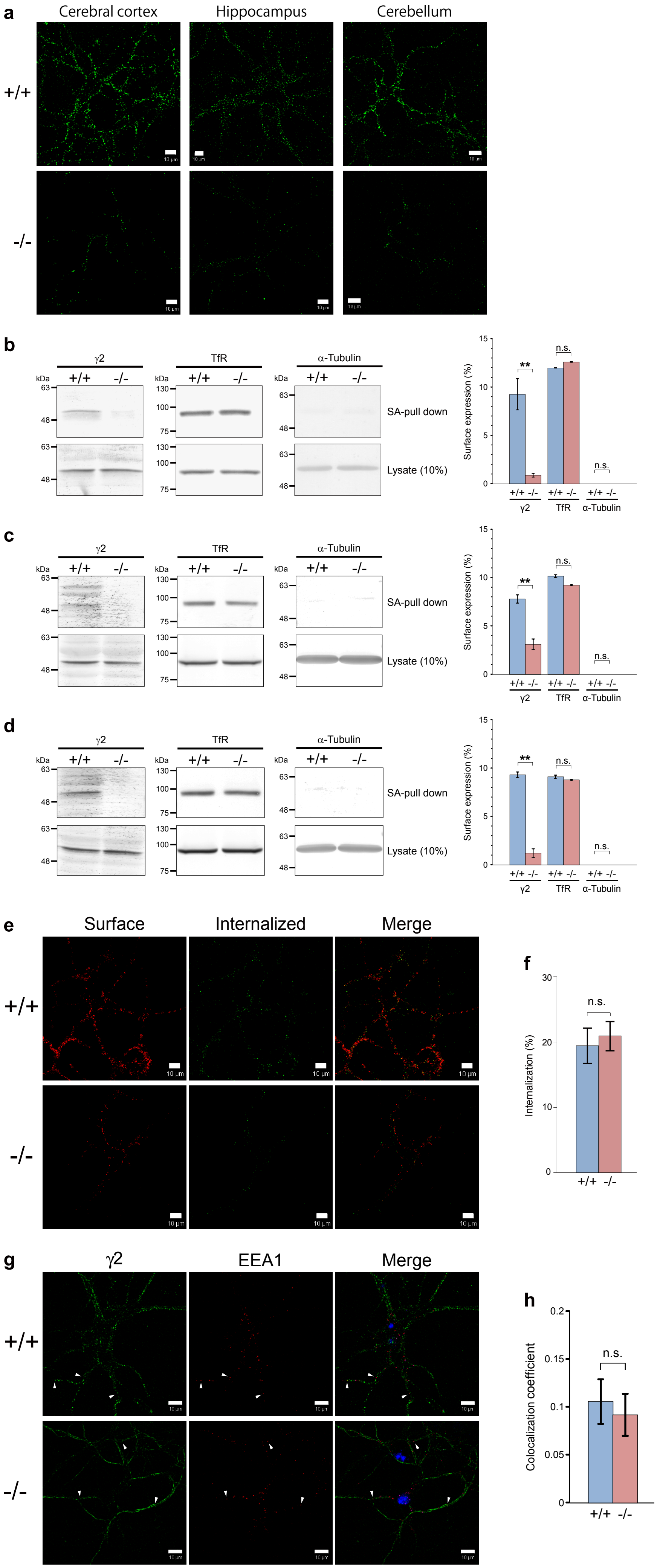
a
Cerebral cortex
Hippocampus
Cerebellum
+/+
-/-
b
γ2
TfR
α-Tubulin
kDa
+/+ -/-
SA-pull down
Lysate (10%)
Surface expression (%)
**
n.s.
γ2
TfR
α-Tubulin
c
d
e
Surface
Internalized
Merge
f
Internalization (%)
n.s.
g
γ2
EEA1
Merge
h
Colocalization coefficient
n.s.

Supplementary Figure 3 | Decreased surface expression of $GABA_A$Rs in *PX-RICS*$^{-/-}$ neurons

(**a**) Surface labelling of γ2 in *PX-RICS*$^{+/+}$ and *PX-RICS*$^{-/-}$ cortical, hippocampal or cerebellar granule neurons. Scale bars, 10 μm. (**b-d**) The amounts of surface and total γ2, transferrin receptor (TfR), α-tubulin in *PX-RICS*$^{+/+}$ and *PX-RICS*$^{-/-}$ cortical (**b**), hippocampal (**c**) and cerebellar granule (**d**) neurons were evaluated with the surface biotinylation assay. TfR and α-tubulin were used as positive and negative controls, respectively. SA-pulldown refers to the surface-expressed population that was biotinylated and separated by streptavidin (SA) beads. Lysates (10%) denotes that 10% of the lysate used in the pulldown was loaded. Surface expression was quantified by measuring the band intensity of the surface and total proteins (right bar graphs). Data were obtained from 3 independent experiments. (**e-h**) No significant difference in γ2 endocytic activity between genotypes. (**e**) The γ2 population that remained on the neuronal surface (red) was stained under non-permeabilized conditions, and the internalized population (green) was stained after permeabilization. Scale bars, 10 μm. (**f**) Quantitative analysis of surface-expressed and intracellular populations. n = 10 (*PX-RICS*$^{+/+}$) and 10 (*PX-RICS*$^{-/-}$). (**g**) Double immunofluorescent staining of γ2 (green) and EEA1 (red). The white arrowheads indicate the representative colocalization signals. Cell nuclei were visualized with TO-PRO-3 (blue). Scale bars, 10 μm. (**h**) Quantitative analysis of the colocalization signals. n = 10 (*PX-RICS*$^{+/+}$) and 10 (*PX-RICS*$^{-/-}$). Data are represented as means ± s.e.m. n.s.; not significant, $^{**}P<0.01$ (unpaired two-tailed Student's *t*-test).

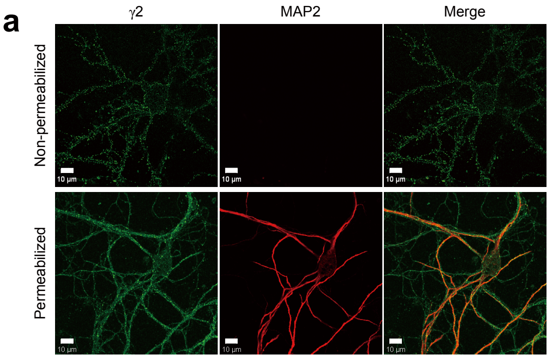

l

		Calreticulin (ER)	Sec23 (ERES)	ERGIC53 (ERGIC)	GM130 (cis-Golgi)	Syntaxin 6 (TGN)
Soma	Intracellular γ2 PX-RICS	○	○	○	×	×
	Intracellular γ2 GABARAP	○	○	○	×	×
Dendrite	Intracellular γ2 PX-RICS	○	○	○	×	○
	Intracellular γ2 GABARAP	○	○	○	×	○

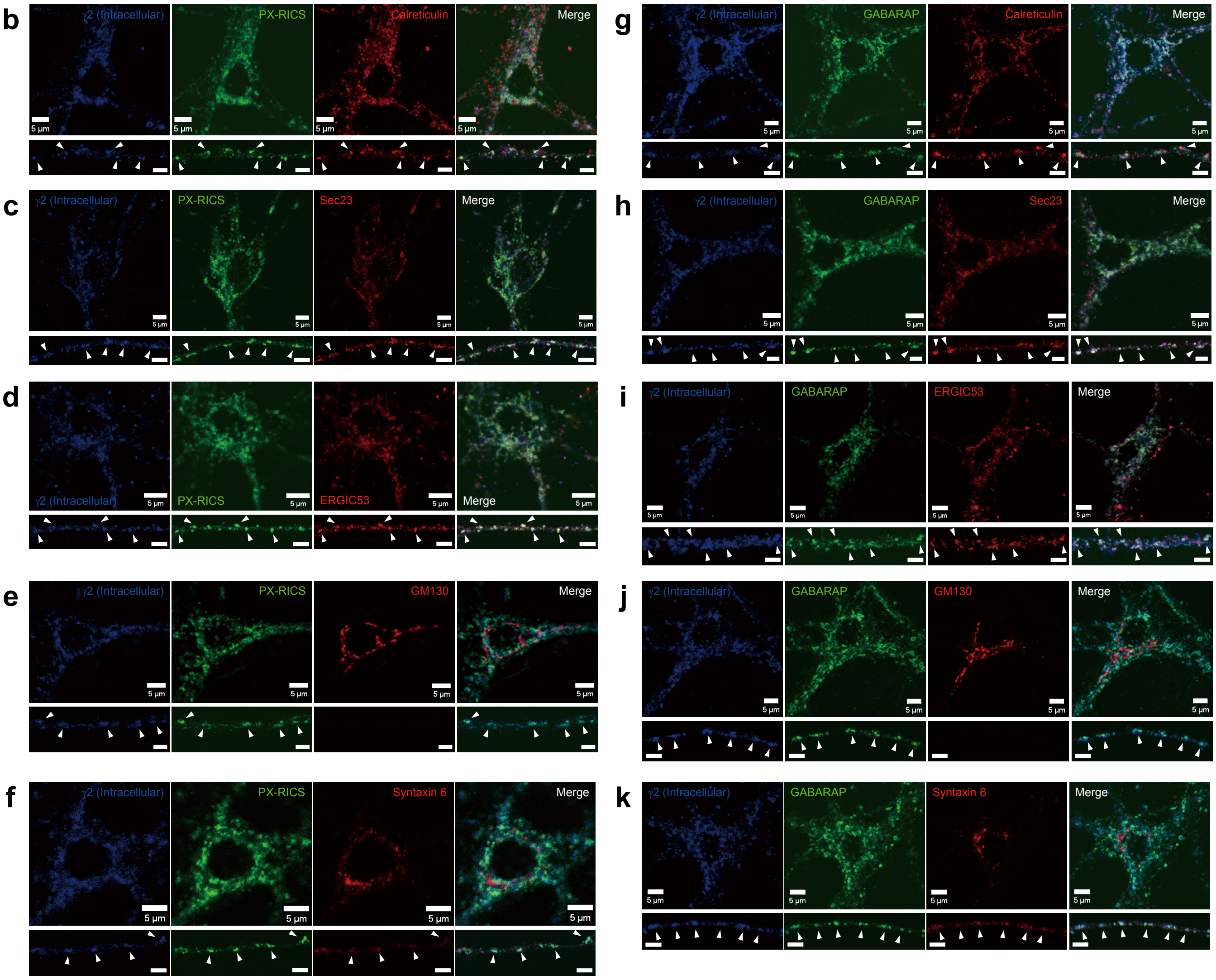

Supplementary Figure 4 | Intracellular γ2, GABARAP and PX-RICS are localized in the somatodendritic secretory pathway in cortical neurons

(**a**) Confirmation of the validity of surface labelling. γ2 (green) and MAP2 (red) staining under non-permeabilized and permeabilized conditions. Anti-γ2 antibody recognizes the N-terminal extracellular region of the γ2 subunits. In the non-permeabilized condition, γ2 staining appeared predominantly as punctate patterns along dendrites and the soma, whereas intracellular protein MAP2 showed minimal staining. In the permeabilized condition, however, markedly greater diffuse somatic and dendritic γ2 staining was observed, in addition to punctate staining. This result shows that our non-permeabilized condition stains surface-expressed γ2 exclusively and that subsequent permeabilization reveals the intracellular population. Scale bars, 10 μm. (**b**-**l**) Intracellular γ2 (blue) and PX-RICS (green, **b**-**f**) or GABARAP (green, **g**-**k**) were co-immunostained with antibodies against organelle-specific proteins (red), calreticulin (ER) (**b**,**g**), Sec23 (ERES) (**c**,**h**), ERGIC53 (ERGIC) (**d**,**i**), GM130 (*cis*-Golgi) (**e**,**j**), syntaxin 6 (TGN) (**f**,**k**). Representative images of the soma and dendrites are shown. Colocalization data are arranged in **l**. ○; colocalized, ×; not colocalized. In the soma, intracellular γ2, PX-RICS and GABARAP were colocalized with markers for the early secretory pathway calreticulin, Sec23 and ERGIC53 but not with syntaxin 6, a marker for the late secretory pathway. In dendrites, intracellular γ2, PX-RICS and GABARAP were also colocalized with syntaxin 6, in addition to calreticulin, Sec23 and ERGIC53 (white arrowheads). Scale bars, 5 μm.

Fig. 4b

Soma

Dendrite

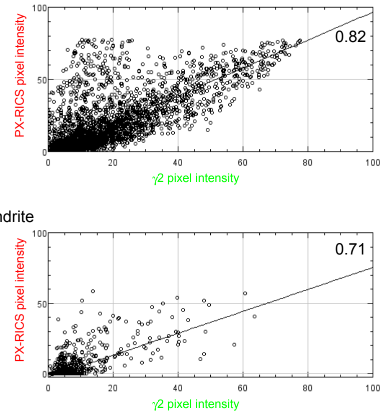

Fig. 4c

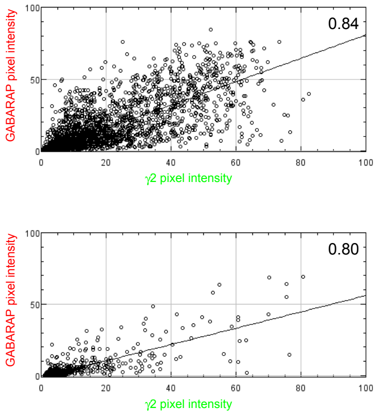

Fig. 4d

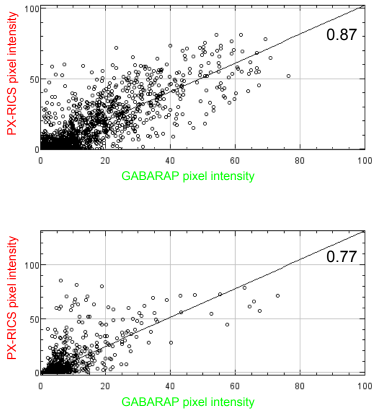

Fig. 4g

Soma

Dendrite

Soma

Dendrite

Soma

Dendrite

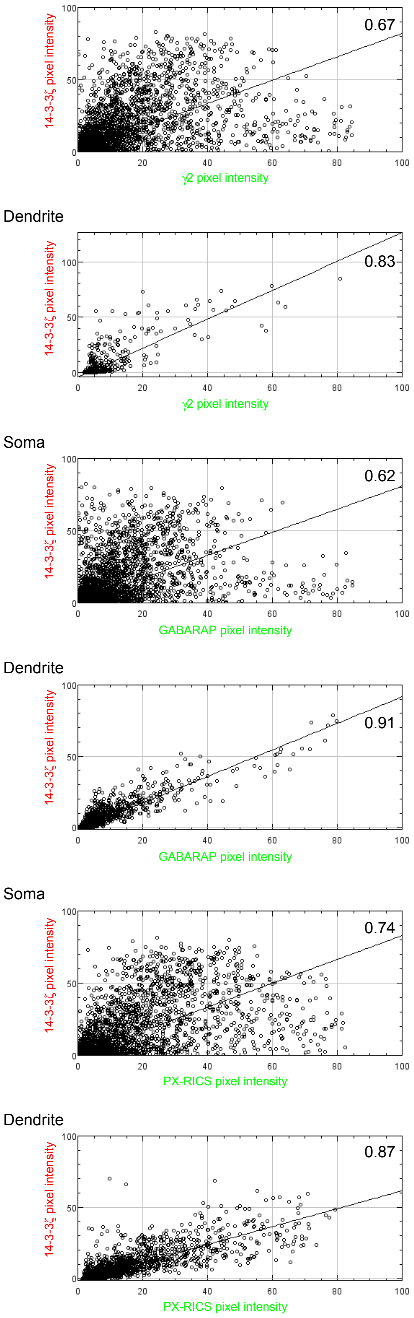

Fig. 4h

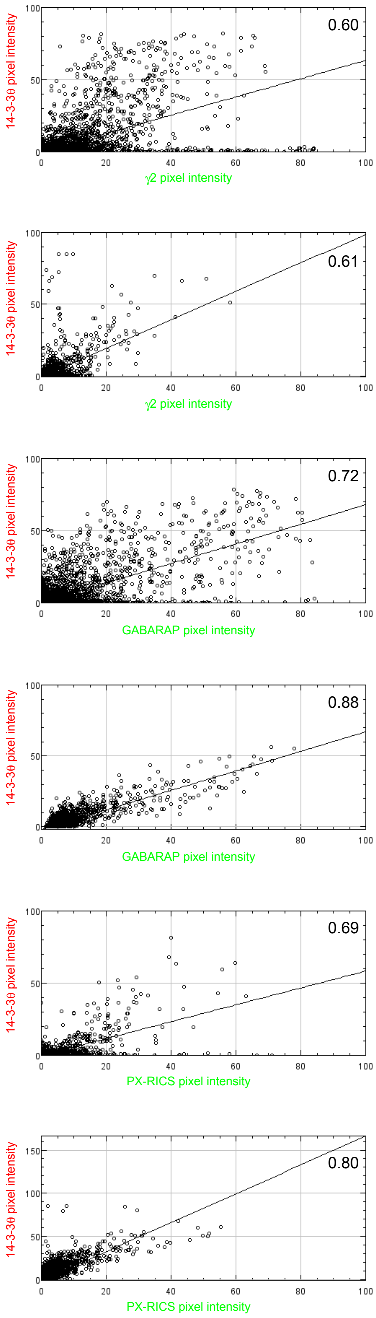

Fig. 4i

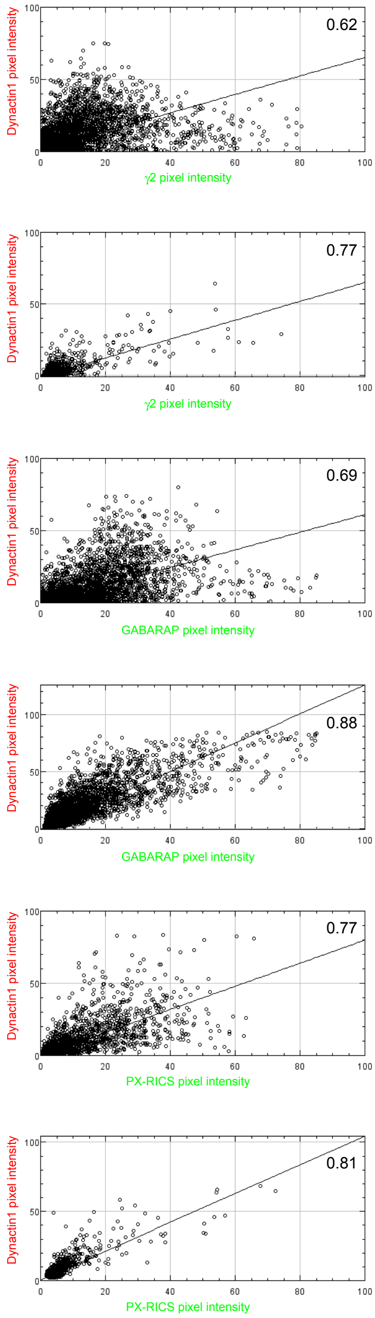

Supplementary Figure 5 | Quantitative analysis of the colocalization signals in Figure 4

The quantification results are expressed as the correlation plots, each of which shows green pixel intensity in a horizontal axis and red pixel intensity in a vertical axis. The correlation coefficient is shown in the upper right corner of each plot.

Supplementary Fig. 4b

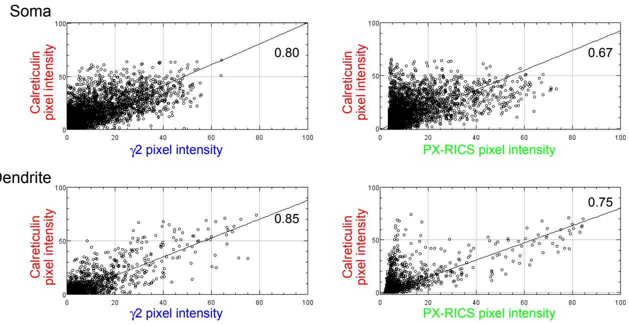

Supplementary Fig. 4c

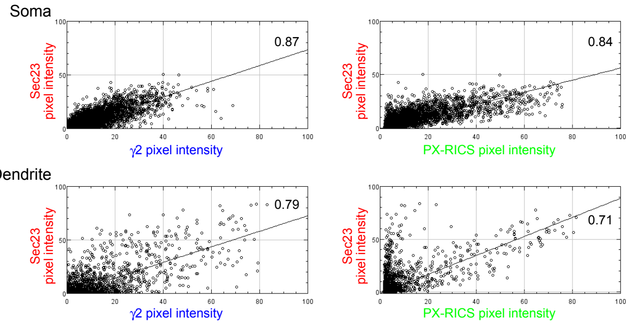

Supplementary Fig. 4d

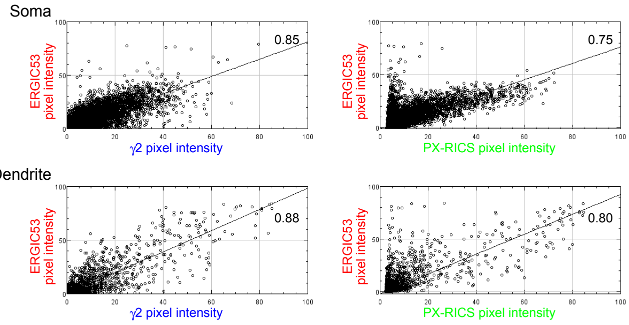

Supplementary Fig. 4e

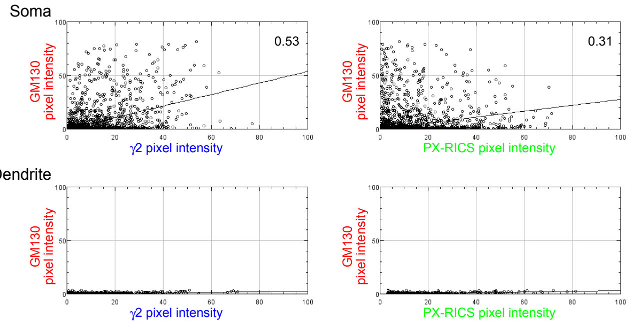

Supplementary Fig. 4f

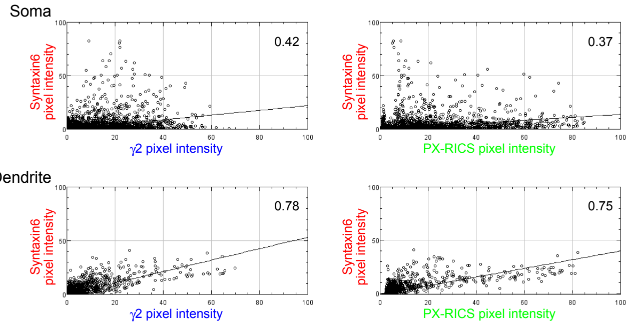

Supplementary Fig. 4g

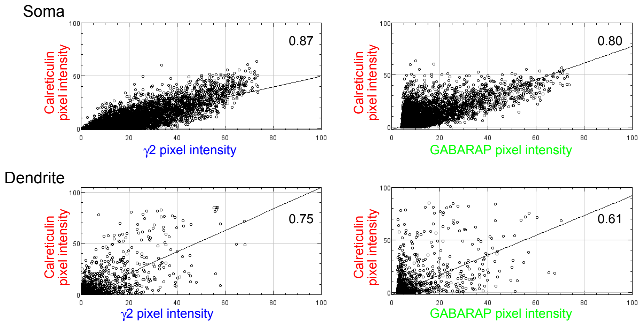

Supplementary Fig. 4h

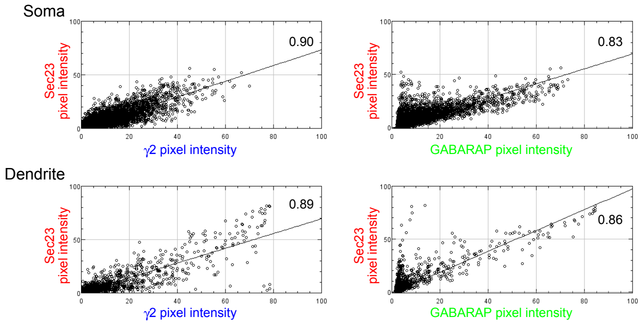

Supplementary Fig. 4i

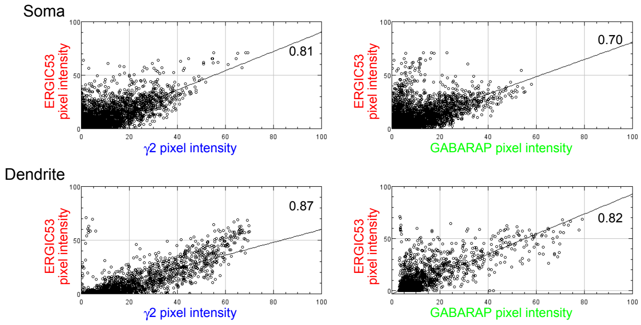

Supplementary Fig. 4j

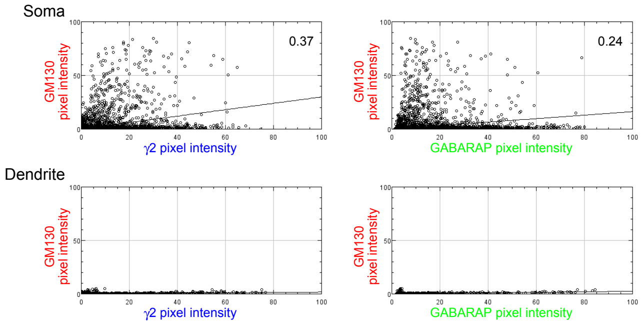

Supplementary Fig. 4k

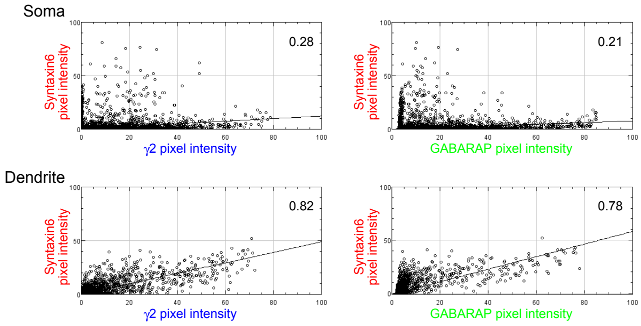

Supplementary Figure 6 | Quantitative analysis of the colocalization signals in Supplementary Figure 4

The quantification results are expressed as the correlation plots. A horizontal axis shows blue or green pixel intensity and a vertical axis red pixel intensity in each plot. The correlation coefficient is shown in the upper right corner of each plot. Virtually no fluorescent signals for GM130 were detected in dendrites and thus the correlation coefficients for γ2/GM130, PX-RICS/GM130 and GABARAP/GM130 pairs were not determined. The quantification result is consistent with the conclusion from our visual observation summarized in Supplementary Fig. 4l.

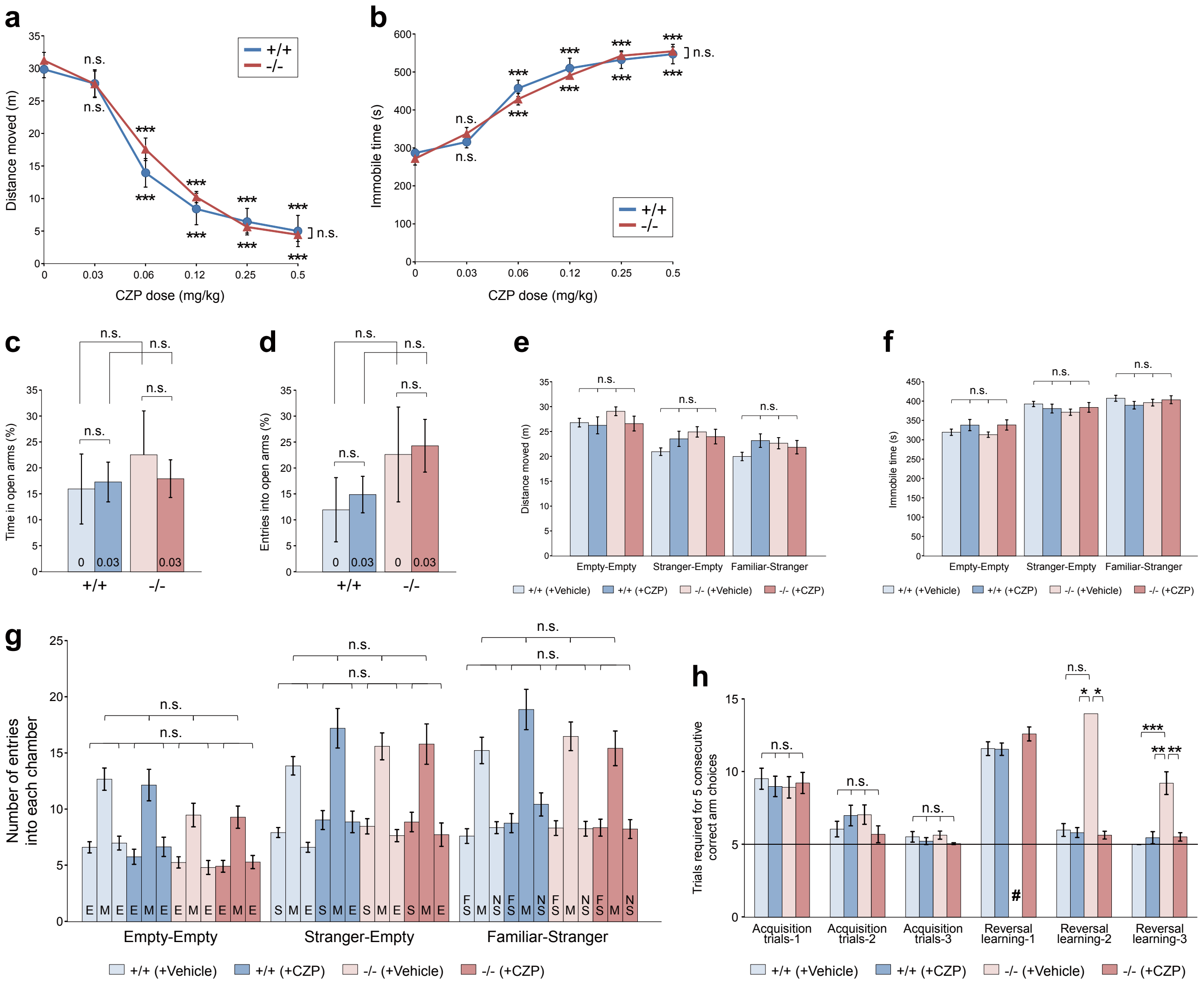

a
Distance moved (m)
CZP dose (mg/kg)
+/+
-/-
n.s.
***
b
Immobile time (s)
CZP dose (mg/kg)
+/+
-/-
c
Time in open arms (%)
d
Entries into open arms (%)
e
Distance moved (m)
Empty-Empty
Stranger-Empty
Familiar-Stranger
+/+ (+Vehicle)
+/+ (+CZP)
-/- (+Vehicle)
-/- (+CZP)
f
Immobile time (s)
g
Number of entries into each chamber
h
Trials required for 5 consecutive correct arm choices
Acquisition trials-1
Acquisition trials-2
Acquisition trials-3
Reversal learning-1
Reversal learning-2
Reversal learning-3

Supplementary Figure 7 | A low dose of CZP has a therapeutic effect on impaired social interaction and inflexible behaviour in *PX-RICS*$^{-/-}$ mice

(**a**,**b**) Locomotor activity test in CZP-injected mice. A dose-dependent decrease in distance moved (**a**) and increase in immobile time (**b**) were observed, but injection of 0.03 mg/kg CZP caused no significant sedative effects. Comparisons with vehicle-treated mice in each genotype and comparisons between genotypes are shown. (**c**,**d**) Elevated plus-maze test. The time spent in open arms (**c**) and the number of entries into open arms (**d**) are shown. No significant anxiolytic effect was observed in mice treated with 0.03 mg/kg CZP. (**e**-**g**) Three-chamber social interaction test. Both vehicle- and CZP-treated mice exhibited comparable levels of locomotor (**e**,**f**) and exploration (**g**) activities in each session. (**h**) Water T-maze test. The ability of CZP-treated *PX-RICS*$^{-/-}$ mice to achieve 5 consecutive correct arm choices was indistinguishable from those of vehicle- or CZP-treated *PX-RICS*$^{+/+}$ mice. As denoted by #, no vehicle-treated *PX-RICS*$^{-/-}$ mice could achieve 5 consecutive correct arm choices on day 1 of reversal learning (Reversal learning-1), and the data were removed from two-way ANOVA. Refer to the statistical analysis data in Supplementary Table 1. Data are represented as means ± s.e.m. n.s.; not significant, **P*<0.05, ***P*<0.01, ****P*<0.001. One-way ANOVA with Tukey's *post hoc* test (**a**-**g**) and two-way ANOVA with Bonferroni's *post hoc* test (**h**).

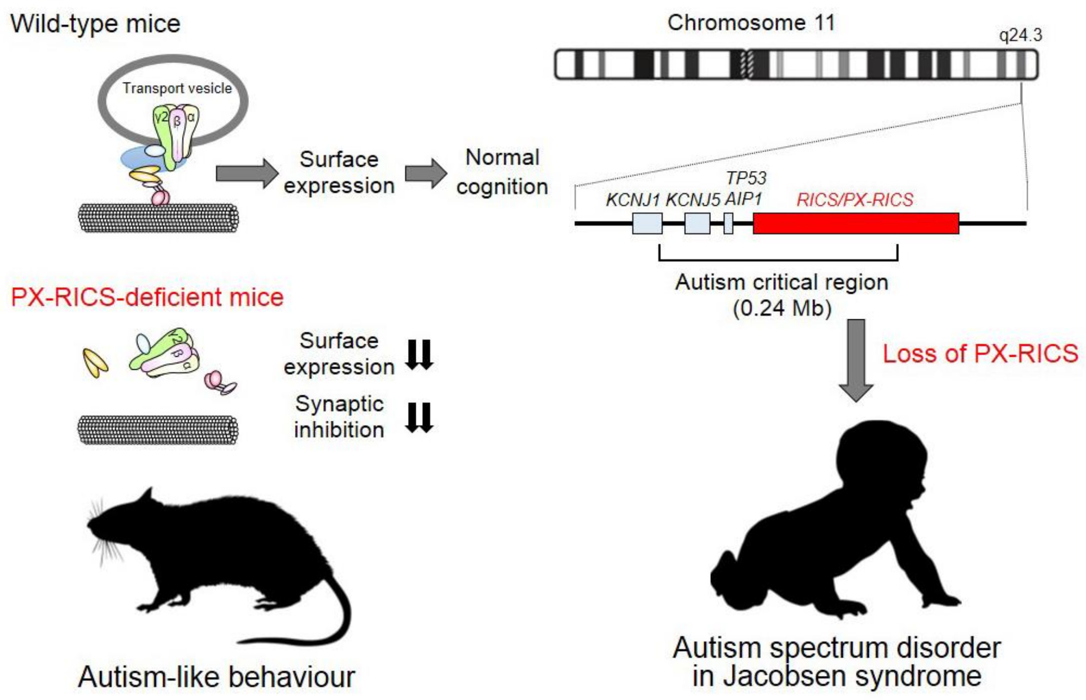

Supplementary Figure 8 | *PX-RICS*-deficient mice recapitulate ASD in patients with Jacobsen syndrome

PX-RICS disruption in mice causes behavioural impairments characteristic of ASD, most likely due to the dysfunction of postsynaptic $GABA_AR$ trafficking mediated by the PX-RICS/GABARAP/14-3-3 adaptor complex and a resulting decrease in GABA-mediated inhibitory neurotransmission (left). Jacobsen syndrome patients with ASD share the loss of four genes on chromosome 11q24.3, including *PX-RICS*. Our findings demonstrate a critical role of PX-RICS in the cognitive functioning of the brain and strongly support the notion that *PX-RICS* is a gene responsible for ASD in Jacobsen syndrome (right).

Fig. 4a

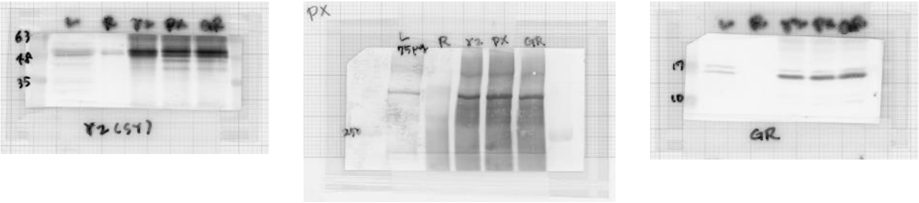

Fig. 4e

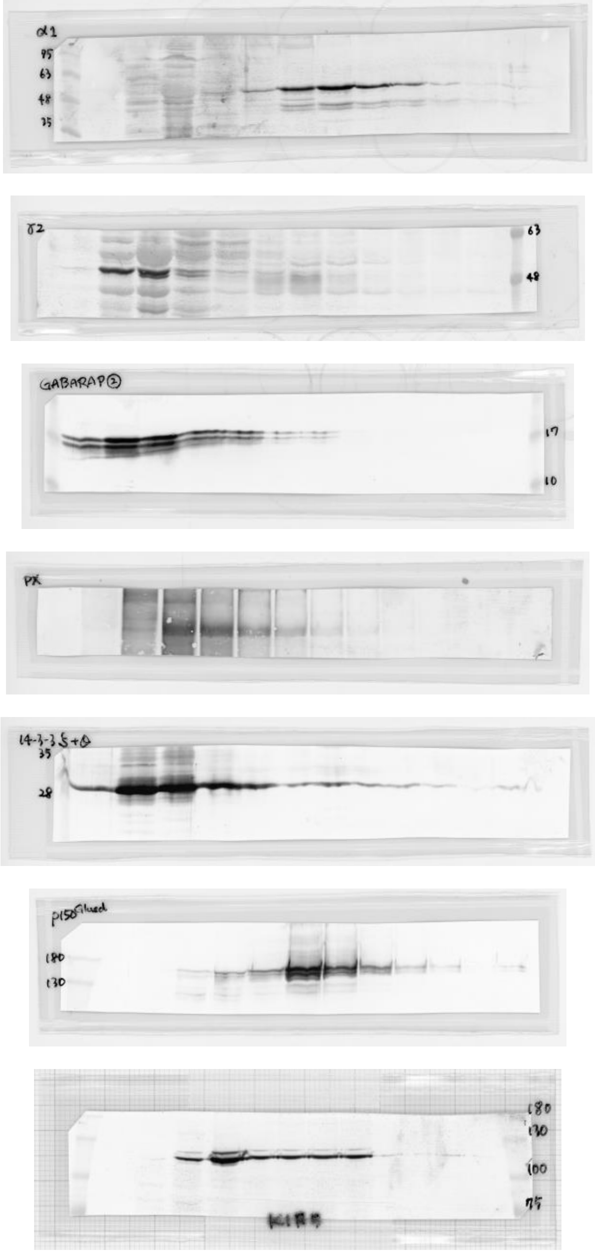

Fig. 4f

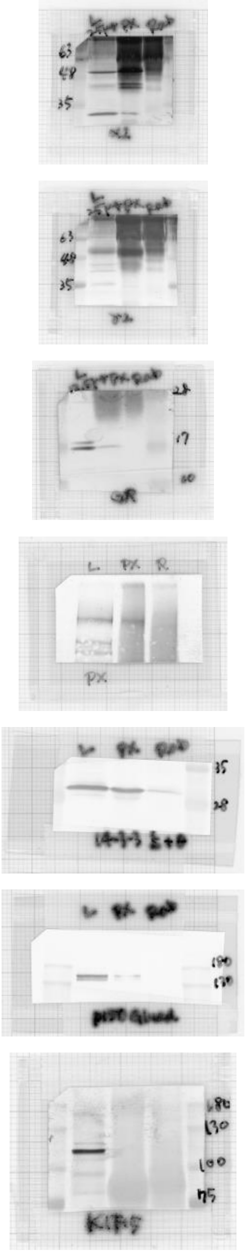

Supplementary Figure 9 | Full-size scans of Western blots shown in Figure 4

Uncropped blots presented in Figure 4a, 4e and 4f are shown.

Supplementary Fig. 3b

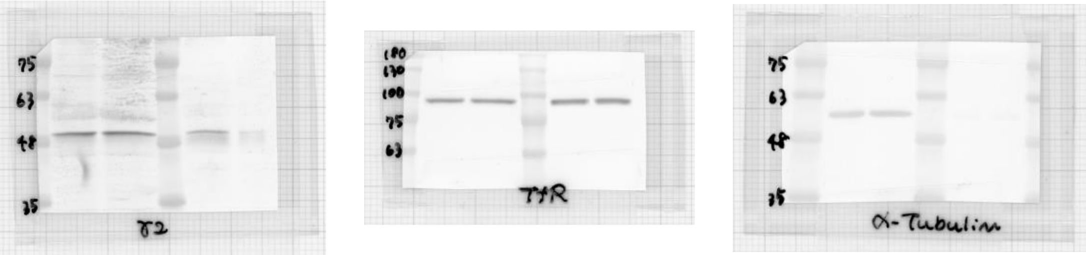

Supplementary Fig. 3c

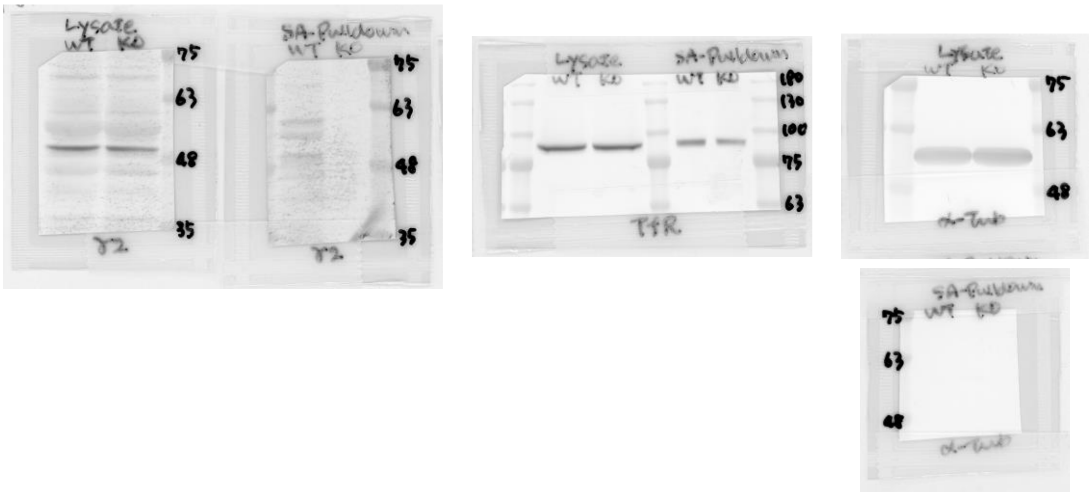

Supplementary Fig. 3d

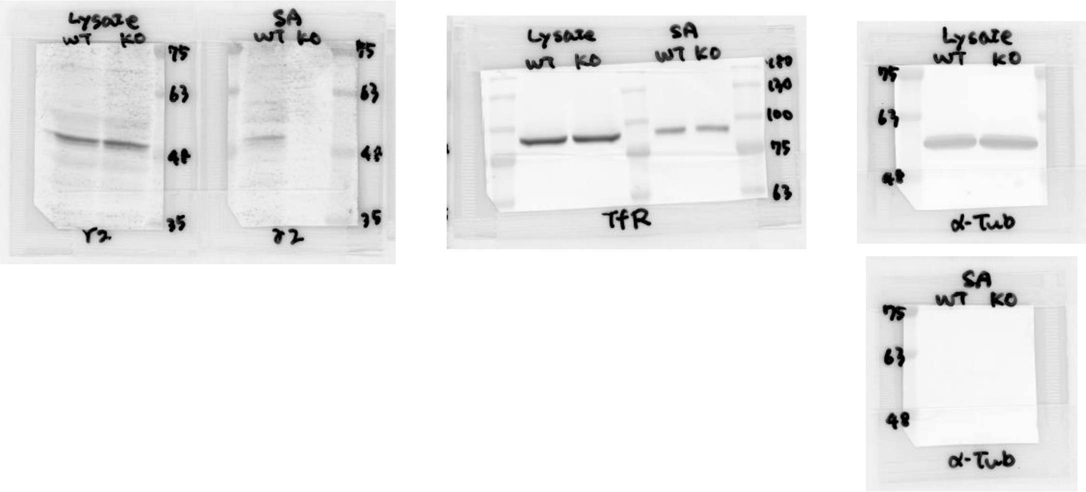

Supplementary Figure 10 | Full-size scans of Western blots shown in Supplementary Figure 3

Uncropped blots presented in Supplementary Figure 3b, 3c and 3d are shown.

Supplementary Table 1. Summary of statistical analysis for the behavioural data

Autism-related behavioural tests

Behavioural test	Number of mice	Measurement	Genotype	Treatment	Mean	s.e.m.	Statistical test	F value	*p* value	*post hoc* test	Comparison	*p* value	Figure
Three-chamber social interaction test	+/+=13 +/-=15 -/-=14	Time in close interaction (Empty-Empty)	+/+	Empty (Left)	90.42	9.85	One-way ANOVA	0.8991	0.4861 n.s.	-	-	-	1a
				Empty (Right)	95.31	10.02							
			+/-	Empty (Left)	84.60	7.38							
				Empty (Right)	104.63	6.73							
			-/-	Empty (Left)	90.79	10.36							
				Empty (Right)	106.07	10.41							
		Time in close interaction (Stranger-Empty)	+/+	Stranger	211.42	14.08	One-way ANOVA	25.88	< 0.0001	Tukey's	Stranger vs. Empty	< 0.0001	
				Empty	91.73	10.69							
			+/-	Stranger	200.53	14.08						< 0.0001	
				Empty	85.67	11.79							
			-/-	Stranger	194.32	14.63						< 0.0001	
				Empty	75.93	10.12							
		Time in close interaction (Familiar-Stranger)	+/+	Familiar stranger	115.19	13.56	One-way ANOVA	5.867	0.0001	Tukey's	Familiar stranger vs. Novel stranger	0.0080	
				Novel stranger	208.50	25.99							
			+/-	Familiar stranger	96.00	11.79						0.1060 n.s.	
				Novel stranger	159.67	6.58							
			-/-	Familiar stranger	164.50	26.91						0.1642 n.s.	
				Novel stranger	103.57	15.49							
		Preference index (control)	+/+	Empty(Right) -Empty(Left)	1.56	4.22	One-way ANOVA	0.3120	0.7338 n.s.	-	-	-	1b
			+/-		5.47	2.32							
			-/-		3.82	3.89							
		Preference index (social preference)	+/+	Stranger-Empty	19.96	2.98	One-way ANOVA	0.1126	0.8938 n.s.	-	-	-	
			+/-		20.30	3.27							
			-/-		22.00	3.38							
		Preference index (social novelty)	+/+	Novel stranger -Familiar stranger	12.52	4.54	One-way ANOVA	10.520	0.0002	Tukey's	+/+ vs. -/-	0.0014	
			+/-		13.60	3.04					+/- vs. -/-	0.0005	
			-/-		-9.90	4.69					+/+ vs. +/-	0.9810 n.s.	
		Time in chamber (Empty-Empty)	+/+	Empty (Left)	237.88	15.27	One-way ANOVA	17.6	< 0.0001	Tukey's	Empty (Left) vs. Middle	< 0.0001	S1b
				Middle	131.35	11.23					Empty (Right) vs. Middle	< 0.0001	
				Empty (Right)	230.77	15.29					Empty (Left) vs. Empty (Right)	> 0.9999 n.s.	
			+/-	Empty (Left)	215.63	11.17					Empty (Left) vs. Middle	< 0.0001	
				Middle	123.43	9.38					Empty (Right) vs. Middle	< 0.0001	
				Empty (Right)	260.93	10.45					Empty (Left) vs. Empty (Right)	0.2489 n.s.	
			-/-	Empty (Left)	218.68	13.43					Empty (Left) vs. Middle	0.0002	
				Middle	129.93	16.53					Empty (Right) vs. Middle	< 0.0001	
				Empty (Right)	251.39	16.30					Empty (Left) vs. Empty (Right)	0.7235 n.s.	
		Time in chamber (Stranger-Empty)	+/+	Stranger	320.31	12.22	One-way ANOVA	87.65	< 0.0001	Tukey's	Stranger vs. Middle	< 0.0001	
				Middle	74.04	7.89					Empty vs. Middle	< 0.0001	
				Empty	205.65	13.59					Stranger vs. Empty	< 0.0001	
			+/-	Stranger	333.00	13.47					Stranger vs. Middle	< 0.0001	
				Middle	67.13	4.65					Empty vs. Middle	< 0.0001	
				Empty	199.87	13.38					Stranger vs. Empty	< 0.0001	
			-/-	Stranger	327.82	15.16					Stranger vs. Middle	< 0.0001	
				Middle	77.54	6.91					Empty vs. Middle	< 0.0001	
				Empty	194.64	13.88					Stranger vs. Empty	< 0.0001	
		Time in chamber (Familiar-Stranger)	+/+	Familiar stranger	214.31	24.01	One-way ANOVA	34.68	< 0.0001	Tukey's	Familiar stranger vs. Middle	< 0.0001	
				Middle	59.42	6.45					Novel stranger vs. Middle	< 0.0001	
				Novel stranger	326.27	25.69					Familiar stranger vs. Novel stranger	0.0024	
			+/-	Familiar stranger	210.43	15.20					Familiar stranger vs. Middle	< 0.0001	
				Middle	72.03	6.52					Novel stranger vs. Middle	< 0.0001	
				Novel stranger	317.53	15.19					Familiar stranger vs. Novel stranger	0.0016	
			-/-	Familiar stranger	316.11	26.95					Familiar stranger vs. Middle	< 0.0001	
				Middle	70.79	8.76					Novel stranger vs. Middle	< 0.0001	
				Novel stranger	213.11	25.26					Familiar stranger vs. Novel stranger	0.0047	
		Preference index (control)	+/+	Empty(Right) -Empty(Left)	-0.71	3.13	One-way ANOVA	1.194	0.3138 n.s.	-	-	-	S1c
			+/-		4.82	2.08							
			-/-		3.22	2.55							
		Preference index (social preference)	+/+	Stranger-Empty	11.01	2.37	One-way ANOVA	0.1272	0.8809 n.s.	-	-	-	
			+/-		12.50	2.50							
			-/-		12.69	2.66							
		Preference index (social novelty)	+/+	Novel stranger -Familiar stranger	10.22	4.36	One-way ANOVA	8.360	0.0010	Tukey's	+/+ vs. -/-	0.0035	
			+/-		10.17	2.70					+/- vs. -/-	0.0025	
			-/-		-9.59	4.71					+/+ vs. +/-	> 0.9999 n.s.	
		Number of entries into each chamber	+/+	Empty (Left)	10.77	0.89	One-way ANOVA	1.356	0.2499 n.s.	-	-	-	S1d
				Empty (Right)	10.31	0.87							
			+/-	Empty (Left)	11.13	1.04							
				Empty (Right)	11.53	0.82							
			-/-	Empty (Left)	8.86	0.75							
				Empty (Right)	9.86	0.56							
			+/+	Stranger	9.77	0.98	One-way ANOVA	1.310	0.2683 n.s.	-	-	-	
				Empty	8.77	0.77							
			+/-	Stranger	11.13	1.03							
				Empty	10.87	1.09							
			-/-	Stranger	11.71	1.13							
				Empty	11.79	0.98							
			+/+	Familiar stranger	7.69	0.83	One-way ANOVA	2.516	0.0365	Tukey's	in all combinations	0.0653- > 0.9999 n.s.	
				Novel stranger	8.23	0.83							
			+/-	Familiar stranger	11.07	0.96							
				Novel stranger	11.53	1.03							
			-/-	Familiar stranger	10.14	0.86							
				Novel stranger	10.29	1.16							
			+/+	Middle (Empty-Empty)	20.31	1.52	One-way ANOVA	1.678	0.2000 n.s.	-	-	-	
			+/-		21.80	1.78							
			-/-		17.93	1.16							
			+/+	Middle (Stranger-Empty)	17.69	1.65	One-way ANOVA	1.784	0.1813 n.s.	-	-	-	
			+/-		21.53	2.11							
			-/-		22.93	2.09							

			+/+	Middle (Familiar-Stranger)	16.08	1.25	One-way ANOVA	2.655	0.0833 n.s.	-	-	-	
			+/-		22.07	1.94							
			-/-		20.07	1.99							
		Distance moved	+/+	Empty-Empty	31.42	1.31	One-way ANOVA	1.036	0.3645 n.s.	-	-	-	S1e
			+/-		34.09	1.83							
			-/-		34.08	1.12							
			+/+	Stranger-Empty	24.84	1.18	One-way ANOVA	2.460	0.0990 n.s.	-	-	-	
			+/-		27.69	1.45							
			-/-		29.61	1.65							
			+/+	Familiar-Stranger	21.94	1.36	One-way ANOVA	2.376	0.1063 n.s.	-	-	-	
			+/-		26.28	1.41							
			-/-		25.57	1.66							
		Immobile time	+/+	Empty-Empty	304.65	9.29	One-way ANOVA	0.3456	0.7099 n.s.	-	-	-	S1f
			+/-		290.77	13.27							
			-/-		298.68	12.06							
			+/+	Stranger-Empty	378.54	10.48	One-way ANOVA	1.180	0.3180 n.s.	-	-	-	
			+/-		360.73	11.79							
			-/-		351.82	14.00							
			+/+	Familiar-Stranger	396.27	12.16	One-way ANOVA	0.9070	0.4121 n.s.	-	-	-	
			+/-		373.00	11.64							
			-/-		389.93	14.27							
Dyadic social interaction test	+/+=15 +/-=16 -/-=16	Time spent in social activities	+/+	-	85.79	6.42	One-way ANOVA	28.14	< 0.0001	Tukey's	+/+ vs. +/-	0.0062	1c
			+/-		64.18	4.41					+/+ vs. -/-	< 0.0001	
			-/-		36.09	2.69					+/- vs. -/-	0.0003	
		Number of sniffing	+/+	-	41.60	3.22	One-way ANOVA	8.942	0.0006	Tukey's	+/+ vs. +/-	0.0030	1d
			+/-		29.31	2.28					+/+ vs. -/-	0.0011	
			-/-		28.13	1.79					+/- vs. -/-	0.9370 n.s.	
		Time spent in social proximity	+/+	-	63.78	4.17	One-way ANOVA	30.24	< 0.0001	Tukey's	+/+ vs. +/-	0.0224	1e
			+/-		48.91	4.43					+/+ vs. -/-	< 0.0001	
			-/-		22.56	2.52					+/- vs. -/-	< 0.0001	
		Successful interaction	+/+	Approach: Test ⟶ Stimulator	88.39	2.75	Two-way ANOVA	Genotype 21.78 Treatment 105.3 Interaction [illegible]	Genotype < 0.0001 Treatment < 0.0001 Interaction [illegible]	Bonferroni's	+/+ vs. +/-	0.2470 n.s.	1f
			+/-		75.45	2.72					+/+ vs. -/-	0.0015	
			-/-		66.82	3.91					+/- vs. -/-	> 0.9999 n.s.	
			+/+	Approach: Stimulator ⟶ Test	61.41	4.36					+/+ vs. +/-	0.0036	
			+/-		41.16	4.54					+/+ vs. -/-	< 0.0001	
			-/-		34.53	3.61					+/- vs. -/-	> 0.9999 n.s.	
Tube test	+/+=19 +/-=20 [illegible]	Winning rate	+/+ vs. +/-	-	61.29 (+/+ win)		Two-tailed binomial test	-	0.0352	-	-	-	1g
			+/+ vs. -/-		93.55 (+/+ win)				< 0.0001				
			+/- vs. -/-		68.57 (+/- win)				0.0002				
Olfactory habituation/ dishabituation test	+/+=15 +/-=15 -/-=15	Time spent sniffing	+/+	Water-1	2.28	0.69	Two-way ANOVA	Genotype 32.70 Treatment 104.3 Interaction 6.742	Genotype < 0.0001 Treatment < 0.0001 Interaction < 0.0001	Bonferroni's	+/+ vs. +/-	> 0.9999 n.s.	S1g
			+/-		3.28	0.65					+/+ vs. -/-	> 0.9999 n.s.	
			-/-		1.82	0.48					+/- vs. -/-	> 0.9999 n.s.	
			+/+	Water-2	0.72	0.27					+/+ vs. +/-	> 0.9999 n.s.	
			+/-		0.74	0.23					+/+ vs. -/-	> 0.9999 n.s.	
			-/-		0.78	0.40					+/- vs. -/-	> 0.9999 n.s.	
			+/+	Water-3	0.29	0.14					+/+ vs. +/-	> 0.9999 n.s.	
			+/-		0.29	0.13					+/+ vs. -/-	> 0.9999 n.s.	
			-/-		0.16	0.09					+/- vs. -/-	> 0.9999 n.s.	
			+/+	Almond-1	3.31	0.82					+/+ vs. +/-	> 0.9999 n.s.	
			+/-		3.93	0.58					+/+ vs. -/-	> 0.9999 n.s.	
			-/-		3.33	0.52					+/- vs. -/-	> 0.9999 n.s.	
			+/+	Almond-2	0.25	0.14					+/+ vs. +/-	> 0.9999 n.s.	
			+/-		0.56	0.19					+/+ vs. -/-	> 0.9999 n.s.	
			-/-		0.48	0.31					+/- vs. -/-	> 0.9999 n.s.	
			+/+	Almond-3	0.08	0.06					+/+ vs. +/-	> 0.9999 n.s.	
			+/-		0.00	0.00					+/+ vs. -/-	> 0.9999 n.s.	
			-/-		0.08	0.06					+/- vs. -/-	> 0.9999 n.s.	
			+/+	Banana-1	3.60	0.59					+/+ vs. +/-	> 0.9999 n.s.	
			+/-		4.07	0.55					+/+ vs. -/-	> 0.9999 n.s.	
			-/-		2.57	0.42					+/- vs. -/-	> 0.9999 n.s.	
			+/+	Banana-2	0.76	0.38					+/+ vs. +/-	> 0.9999 n.s.	
			+/-		0.79	0.21					+/+ vs. -/-	> 0.9999 n.s.	
			-/-		0.33	0.13					+/- vs. -/-	> 0.9999 n.s.	
			+/+	Banana-3	0.31	0.15					+/+ vs. +/-	> 0.9999 n.s.	
			+/-		0.03	0.03					+/+ vs. -/-	> 0.9999 n.s.	
			-/-		0.00	0.00					+/- vs. -/-	> 0.9999 n.s.	
			+/+	Urine1-1	13.05	1.37					+/+ vs. +/-	0.0949 n.s.	
			+/-		10.02	1.31					+/+ vs. -/-	< 0.0001	
			-/-		4.15	1.09					+/- vs. -/-	< 0.0001	
			+/+	Urine1-2	2.25	0.69					+/+ vs. +/-	> 0.9999 n.s.	
			+/-		1.83	0.75					+/+ vs. -/-	> 0.9999 n.s.	
			-/-		0.73	0.37					+/- vs. -/-	> 0.9999 n.s.	
			+/+	Urine1-3	0.34	0.20					+/+ vs. +/-	> 0.9999 n.s.	
			+/-		0.86	0.34					+/+ vs. -/-	> 0.9999 n.s.	
			-/-		0.35	0.19					+/- vs. -/-	> 0.9999 n.s.	
			+/+	Urine2-1	10.19	1.09					+/+ vs. +/-	< 0.0001	
			+/-		6.02	0.79					+/+ vs. -/-	< 0.0001	
			-/-		1.93	0.50					+/- vs. -/-	0.0001	
			+/+	Urine2-2	1.43	0.44					+/+ vs. +/-	> 0.9999 n.s.	
			+/-		0.45	0.28					+/+ vs. -/-	> 0.9999 n.s.	
			-/-		0.40	0.19					+/- vs. -/-	> 0.9999 n.s.	
			+/+	Urine2-3	0.15	0.09					+/+ vs. +/-	> 0.9999 n.s.	
			+/-		0.49	0.32					+/+ vs. -/-	> 0.9999 n.s.	
			-/-		0.02	0.02					+/- vs. -/-	> 0.9999 n.s.	
			+/+	Food-1	12.05	0.92					+/+ vs. +/-	> 0.9999 n.s.	
			+/-		10.71	0.53					+/+ vs. -/-	> 0.9999 n.s.	
			-/-		9.80	0.50					+/- vs. -/-	> 0.9999 n.s.	
			+/+	Food-2	2.11	0.49					+/+ vs. +/-	> 0.9999 n.s.	
			+/-		2.70	0.71					+/+ vs. -/-	> 0.9999 n.s.	
			-/-		1.01	0.43					+/- vs. -/-	> 0.9999 n.s.	
			+/+	Food-3	1.08	0.47					+/+ vs. +/-	> 0.9999 n.s.	
			+/-		0.67	0.28					+/+ vs. -/-	> 0.9999 n.s.	
			-/-		1.20	0.49					+/- vs. -/-	> 0.9999 n.s.	
Ultrasonic vocalizations	+/+=16 +/-=24 -/-=17	Total number of calls	+/+	PND5	175.44	32.37	One-way ANOVA	0.2602	0.7718 n.s.	-	-	-	1h
			+/-		181.96	24.35							
			-/-		203.29	25.24							
			+/+	PND7	238.75	37.70	One-way ANOVA	2.235	0.1168 n.s.	-	-	-	
			+/-		164.13	21.52							
			-/-		163.76	25.76							
			+/+	PND10	176.06	28.95	One-way ANOVA	3.494	0.0374	Tukey's	+/+ vs. +/-	0.2716 n.s.	
			+/-		122.29	25.86					+/+ vs. -/-	0.0286	
			-/-		77.82	12.93					-	-	
			+/+	PND14	115.94	33.94	One-way ANOVA	3.423	0.0398	Tukey's	+/+ vs. +/-	0.2925 n.s.	
			+/-		71.04	15.96					+/+ vs. -/-	0.0305	
			-/-		32.24	11.97					-	-	
			+/+	PND21	2.50	1.30	One-way ANOVA	5.842	0.0050	Tukey's	+/+ vs. +/-	0.2750 n.s.	
			+/-		0.75	0.24					+/+ vs. -/-	0.2267 n.s.	
			-/-		4.53	0.90					-	-	
		Mean duration of calls	+/+	PND5	0.03038	0.00405	One-way ANOVA	2.204	0.1202 n.s.	-	-	-	S2b
			+/-		0.03679	0.00224							
			-/-		0.03899	0.00228							
			+/+	PND7	0.03449	0.00261	One-way ANOVA	3.470	0.0382	Tukey's	+/+ vs. +/-	0.4305 n.s.	
			+/-		0.03049	0.00213					+/+ vs. -/-	0.4384 n.s.	
			-/-		0.03877	0.00209					-	-	
			+/+	PND10	0.03290	0.00209	One-way ANOVA	0.1564	0.8556 n.s.	-	-	-	
			+/-		0.03160	0.00265							
			-/-		0.03073	0.00260							
			+/+	PND14	0.03290	0.00488	One-way ANOVA	1.567	0.2180 n.s.	-	-	-	
			+/-		0.02345	0.00305							
			-/-		0.02307	0.00525							
			+/+	PND21	0.00272	0.00091	One-way [illegible]	2.146	0.1268 n.s.	-	-	-	
			+/-		0.00245	0.00114							

			-/-		0.00545	0.00113	ANOVA						
		Total calling time	+/+	PND5	6.760	1.739	One-way ANOVA	0.2533	0.7772 n.s	-	-	-	S2c
			+/-		7.411	1.320							
			-/-		8.293	1.219							
			+/+	PND7	8.814	1.654	One-way ANOVA	1.644	0.2027 n.s.	-	-	-	
			+/-		5.691	0.965							
			-/-		6.834	1.166							
			+/+	PND10	6.444	1.349	One-way ANOVA	1.680	0.1960 n.s.	-	-	-	
			+/-		5.037	1.498							
			-/-		2.833	0.600							
			+/+	PND14	5.536	2.090	One-way ANOVA	2.813	0.0688 n.s.	-	-	-	
			+/-		2.490	0.702							
			-/-		1.460	0.647							
			+/+	PND21	0.012	0.005	One-way ANOVA	7.187	0.0017	Tukey's	+/+ vs. +/-	0.6403 n.s.	
			+/-		0.005	0.002					+/+ vs. -/-	0.0351	
			-/-		0.031	0.008					-	-	
		Latency to the 1st call	+/+	PND5	32.760	8.697	One-way ANOVA	0.4104	0.6654 n.s.	-	-	-	S2a
			+/-		26.787	6.490							
			-/-		22.850	6.790							
			+/+	PND7	18.709	7.007	One-way ANOVA	3.994	0.0241	Tukey's	+/+ vs. +/-	0.7916 n.s.	
			+/-		12.266	2.092					+/+ vs. -/-	0.1421 n.s.	
			-/-		39.192	11.554					-	-	
			+/+	PND10	26.564	9.173	One-way ANOVA	0.7397	0.4820 n.s.	-	-	-	
			+/-		41.391	9.361							
			-/-		40.264	8.252							
			+/+	PND14	49.899	12.125	One-way ANOVA	6.114	0.0040	Tukey's	+/+ vs. +/-	0.2212 n.s.	
			+/-		104.833	23.358					+/+ vs. -/-	0.0028	
			-/-		172.648	28.196					-	-	
			+/+	PND21	171.594	34.941	One-way ANOVA	6.149	0.0039	Tukey's	+/+ vs. +/-	0.2702 n.s.	
			+/-		232.176	23.275					+/+ vs. -/-	0.1998 n.s.	
			-/-		98.823	26.175					-	-	
		Peak frequency	+/+	PND5	77.61	1.56	One-way ANOVA	0.4143	0.6629 n.s.	-	-	-	S2d
			+/-		77.12	1.19							
			-/-		78.86	1.55							
			+/+	PND7	80.91	1.59	One-way ANOVA	0.4327	0.6510 n.s.	-	-	-	
			+/-		81.08	1.13							
			-/-		82.70	1.73							
			+/+	PND10	79.70	1.85	One-way ANOVA	0.6964	0.5028 n.s.	-	-	-	
			+/-		79.25	0.90							
			-/-		81.42	1.52							
			+/+	PND14	81.21	1.25	One-way ANOVA	1.418	0.2530 n.s.	-	-	-	
			+/-		79.74	1.22							
			-/-		84.01	2.39							
			+/+	PND21	90.51	1.08	One-way ANOVA	2.071	0.1456 n.s.	-	-	-	
			+/-		90.97	2.29							
			-/-		83.86	2.42							
		Peak amplitude	+/+	PND5	-38.756	1.651	One-way ANOVA	2.561	0.0866 n.s.	-	-	-	S2e
			+/-		-36.071	0.720							
			-/-		-35.205	0.982							
			+/+	PND7	-39.646	1.181	One-way ANOVA	2.310	0.1090 n.s.	-	-	-	
			+/-		-40.916	0.967							
			-/-		-37.722	1.112							
			+/+	PND10	-44.615	1.276	One-way ANOVA	2.896	0.0638 n.s.	-	-	-	
			+/-		-42.087	0.940							
			-/-		-40.911	0.912							
			+/+	PND14	-47.823	0.831	One-way ANOVA	1.370	0.2646 n.s.	-	-	-	
			+/-		-48.958	0.829							
			-/-		-46.640	0.986							
			+/+	PND21	-50.905	0.599	One-way ANOVA	0.7211	0.4954 n.s.	-	-	-	
			+/-		-48.111	1.448							
			-/-		-48.797	1.091							
Self-grooming	+/+=16 +/-=16 -/-=15	Time spent self-grooming	+/+	-	55.42	11.79	One-way ANOVA	5.495	0.0074	Tukey's	+/+ vs. +/-	0.7148 n.s.	1i
			+/-		72.62	15.67					+/+ vs. -/-	0.0073	
			-/-		126.57	19.17					+/- vs. -/-	0.0508 n.s.	
Marble burying test	+/+=16 +/-=16 -/-=15	Area of marbles buried	+/+	-	26.06	3.52	One-way ANOVA	15.81	< 0.0001	Tukey's	+/+ vs. +/-	0.0067	1j
			+/-		45.18	5.06					+/+ vs. -/-	< 0.0001	
			-/-		59.85	3.97					+/- vs. -/-	0.0492	
		Number of marbles buried	+/+	-	4.88	0.89	One-way ANOVA	16.47	< 0.0001	Tukey's	+/+ vs. +/-	0.0062	1k
			+/-		9.38	1.08					+/+ vs. -/-	< 0.0001	
			-/-		12.93	0.98					+/- vs. -/-	0.0397	
Water T-maze test	+/+=20 +/-=20 -/-=23	Number of correct trials	+/+	Acquisition trials-1	11.05	0.60	Two-way ANOVA	Genotype 40.48 Treatment 154.1 Interaction 12.40	Genotype < 0.0001 Treatment < 0.0001 Interaction < 0.0001	Bonferroni's	+/+ vs. +/-	> 0.9999 n.s.	1l
			+/-		10.95	0.46					+/+ vs. -/-	> 0.9999 n.s.	
			-/-		11.43	0.49					+/- vs. -/-	> 0.9999 n.s.	
			+/+	Acquisition trials-2	14.05	0.35					+/+ vs. +/-	> 0.9999 n.s.	
			+/-		12.85	0.52					+/+ vs. -/-	> 0.9999 n.s.	
			-/-		13.65	0.22					+/- vs. -/-	> 0.9999 n.s.	
			+/+	Acquisition trials-3	14.95	0.05					+/+ vs. +/-	> 0.9999 n.s.	
			+/-		14.80	0.12					+/+ vs. -/-	> 0.9999 n.s.	
			-/-		15.00	0.00					+/- vs. -/-	> 0.9999 n.s.	
			+/+	Reversal learning-1	5.15	0.79					+/+ vs. +/-	> 0.9999 n.s.	
			+/-		3.65	0.84					+/+ vs. -/-	0.0008	
			-/-		1.43	0.52					+/- vs. -/-	0.9413 n.s.	
			+/+	Reversal learning-2	13.70	0.36					+/+ vs. +/-	> 0.9999 n.s.	
			+/-		12.55	0.79					+/+ vs. -/-	< 0.0001	
			-/-		5.17	0.98					+/- vs. -/-	< 0.0001	
			+/+	Reversal learning-3	14.90	0.07					+/+ vs. +/-	> 0.9999 n.s.	
			+/-		14.85	0.08					+/+ vs. -/-	< 0.0001	
			-/-		10.26	1.04					+/- vs. -/-	< 0.0001	
		Trials required for 5 consecutive correct arm choices	+/+	Acquisition trials-1	9.00	0.63	Two-way ANOVA#	Genotype 4.371 Treatment 32.16 Interaction 1.744	Genotype 0.0136 Treatment < 0.0001 Interaction 0.0885	Bonferroni's	+/+ vs. +/-	> 0.9999 n.s.	S2g
			+/-		9.82	0.70					+/+ vs. -/-	> 0.9999 n.s.	
			-/-		9.62	0.70					+/- vs. -/-	> 0.9999 n.s.	
			+/+	Acquisition trials-2	6.10	0.56					+/+ vs. +/-	> 0.9999 n.s.	
			+/-		7.35	0.61					+/+ vs. -/-	> 0.9999 n.s.	
			-/-		6.30	0.40					+/- vs. -/-	> 0.9999 n.s.	
			+/+	Acquisition trials-3	5.00	0.00					+/+ vs. +/-	> 0.9999 n.s.	
			+/-		5.20	0.20					+/+ vs. -/-	> 0.9999 n.s.	
			-/-		5.00	0.00					+/- vs. -/-	> 0.9999 n.s.	
			+/+	Reversal learning-1	12.70	0.52					+/+ vs. +/-	Not determined#	
			+/-		11.40	0.58					+/+ vs. -/-		
			-/-		-	-					+/- vs. -/-		
			+/+	Reversal learning-2	6.55	0.53					+/+ vs. +/-	> 0.9999 n.s.	
			+/-		7.16	0.56					+/+ vs. -/-	> 0.9999 n.s.	
			-/-		8.43	0.77					+/- vs. -/-	> 0.9999 n.s.	
			+/+	Reversal learning-3	5.05	0.05					+/+ vs. +/-	> 0.9999 n.s.	
			+/-		5.10	0.07					+/+ vs. -/-	0.2434 n.s.	
			-/-		7.40	0.74					+/- vs. -/-	0.3003 n.s.	
		Latency to escape	+/+	Acquisition trials-1	15.92	0.87	Two-way ANOVA	Genotype 32.34 Treatment 109.0 Interaction 7.856	Genotype < 0.0001 Treatment < 0.0001 Interaction < 0.0001	Bonferroni's	+/+ vs. +/-	> 0.9999 n.s.	1m
			+/-		16.38	1.00					+/+ vs. -/-	> 0.9999 n.s.	
			-/-		14.97	0.72					+/- vs. -/-	> 0.9999 n.s.	
			+/+	Acquisition trials-2	9.08	0.76					+/+ vs. +/-	> 0.9999 n.s.	
			+/-		10.66	0.85					+/+ vs. -/-	> 0.9999 n.s.	
			-/-		10.10	0.45					+/- vs. -/-	> 0.9999 n.s.	
			+/+	Acquisition trials-3	5.71	0.15					+/+ vs. +/-	> 0.9999 n.s.	
			+/-		6.06	0.18					+/+ vs. -/-	> 0.9999 n.s.	
			-/-		6.07	0.13					+/- vs. -/-	> 0.9999 n.s.	
			+/+	Reversal learning-1	16.81	1.00					+/+ vs. +/-	> 0.9999 n.s.	
			+/-		18.74	1.37					+/+ vs. -/-	< 0.0001	
			-/-		24.58	1.21					+/- vs. -/-	0.0007	
			+/+	Reversal learning-2	6.13	0.24					+/+ vs. +/-	> 0.9999 n.s.	
			+/-		7.58	1.14					+/+ vs. -/-	< 0.0001	
			-/-		15.83	1.62					+/- vs. -/-	< 0.0001	
			+/+	Reversal	5.14	0.08					+/+ vs. +/-	> 0.9999 n.s.	

			+/-	Reversal learning-3	5.35	0.16					+/+ vs. -/-	0.0013	
			-/-		10.81	1.28					+/- vs. -/-	0.0027	
Accelerating rotarod test	+/+=18<br>+/-=17<br>-/-=17	Latency to fall	+/+	Day 1	87.50	7.73	Two-way ANOVA	Genotype 91.75<br>Treatment 13.93<br>Interaction 0.7360	Genotype < 0.0001<br>Treatment < 0.0001<br>Interaction 0.6596	Bonferroni's	+/+ vs. +/-	> 0.9999 n.s.	2a
			+/-		59.94	9.20					+/+ vs. -/-	< 0.0001	
			-/-		23.47	3.05					+/- vs. -/-	0.3200 n.s.	
			+/+	Day 2	102.39	5.71					+/+ vs. +/-	> 0.9999 n.s.	
			+/-		80.94	8.81					+/+ vs. -/-	< 0.0001	
			-/-		31.82	5.20					+/- vs. -/-	0.0078	
			+/+	Day 3	109.67	9.25					+/+ vs. +/-	> 0.9999 n.s.	
			+/-		94.94	10.38					+/+ vs. -/-	< 0.0001	
			-/-		48.12	5.67					+/- vs. -/-	0.0163	
			+/+	Day 4	123.67	10.69					+/+ vs. +/-	> 0.9999 n.s.	
			+/-		111.53	11.66					+/+ vs. -/-	< 0.0001	
			-/-		49.24	5.21					+/- vs. -/-	< 0.0001	
			+/+	Day 5	131.67	10.23					+/+ vs. +/-	> 0.9999 n.s.	
			+/-		125.65	12.49					+/+ vs. -/-	< 0.0001	
			-/-		52.47	5.75					+/- vs. -/-	< 0.0001	

Since no *PX-RICS*$^{-/-}$ mice could make a 5 consecutive correct arm choice in the reversal learning-1, the corresponding data were removed in the two-way ANOVA.

Behavioural tests of clonazepam-administered mice

Behavioural test	Number of mice	Measurement	Genotype	Treatment	Mean	s.e.m.	Statistical test	F value	*p* value	*post hoc* test	Comparison	*p* value	Figure
Locomotor activity test	+/+=10<br>-/-=10<br>in each dose	Distance moved	+/+	0.00 mg/kg	29.84	1.28	One-way ANOVA	35.69	< 0.0001	Tukey's	+/+ 0.00 vs. 0.03	0.9993 n.s.	S7a
				0.03 mg/kg	27.65	2.14					+/+ 0.00 vs. 0.06	< 0.0001	
				0.06 mg/kg	13.95	2.19					+/+ 0.00 vs. 0.12	< 0.0001	
				0.12 mg/kg	8.39	2.43					+/+ 0.00 vs. 0.25	< 0.0001	
				0.25 mg/kg	6.44	2.04					+/+ 0.00 vs. 0.50	< 0.0001	
				0.50 mg/kg	5.00	2.40					-/- 0.00 vs. 0.03	0.9528n.s.	
			-/-	0.00 mg/kg	31.22	1.23					-/- 0.00 vs. 0.06	< 0.0001	
				0.03 mg/kg	27.60	2.01					-/- 0.00 vs. 0.12	< 0.0001	
				0.06 mg/kg	17.56	1.73					-/- 0.00 vs. 0.25	< 0.0001	
				0.12 mg/kg	10.22	0.87					-/- 0.00 vs. 0.50	< 0.0001	
				0.25 mg/kg	5.62	0.93					+/+ vs. -/-<br>in each dose	> 0.9539 n.s.	
				0.50 mg/kg	4.43	1.02							
		Immobile time	+/+	0.00 mg/kg	286.60	10.27	One-way ANOVA	37.03	< 0.0001	Tukey's	+/+ 0.00 vs. 0.03	0.9913 n.s.	S7b
				0.03 mg/kg	315.65	15.30					+/+ 0.00 vs. 0.06	< 0.0001	
				0.06 mg/kg	457.00	21.34					+/+ 0.00 vs. 0.12	< 0.0001	
				0.12 mg/kg	509.85	26.66					+/+ 0.00 vs. 0.25	< 0.0001	
				0.25 mg/kg	532.45	23.30					+/+ 0.00 vs. 0.50	< 0.0001	
				0.50 mg/kg	547.10	25.61					-/- 0.00 vs. 0.03	0.2864 n.s.	
			-/-	0.00 mg/kg	271.90	17.21					-/- 0.00 vs. 0.06	< 0.0001	
				0.03 mg/kg	337.70	16.07					-/- 0.00 vs. 0.12	< 0.0001	
				0.06 mg/kg	428.30	15.14					-/- 0.00 vs. 0.25	< 0.0001	
				0.12 mg/kg	490.70	9.24					-/- 0.00 vs. 0.50	< 0.0001	
				0.25 mg/kg	542.90	9.98					+/+ vs. -/-<br>in each dose	> 0.9921 n.s.	
				0.50 mg/kg	554.35	11.63							
Elevated plus-maze test	+/+=9<br>-/-=9<br>in each dose	Time in open arms	+/+	0.00 mg/kg	15.98	6.74	One-way ANOVA	0.2270	0.8769 n.s.	Tukey's	+/+ 0.00 vs. 0.03	0.9986 n.s.	S7c
				0.03 mg/kg	17.32	3.83					-/- 0.00 vs. 0.03	0.9479 n.s.	
			-/-	0.00 mg/kg	22.58	8.46					0.00 +/+ vs. -/-	0.8650 n.s.	
				0.03 mg/kg	17.96	3.64					0.03 +/+ vs. -/-	0.9998 n.s.	
		Entries into open arms	+/+	0.00 mg/kg	12.00	6.19	One-way ANOVA	0.8851	0.4592 n.s.	Tukey's	+/+ 0.00 vs. 0.03	0.9877 n.s.	S7d
				0.03 mg/kg	14.92	3.52					-/- 0.00 vs. 0.03	0.9976 n.s.	
			-/-	0.00 mg/kg	22.65	9.14					0.00 +/+ vs. -/-	0.6370 n.s.	
				0.03 mg/kg	24.33	5.08					0.03 +/+ vs. -/-	0.7210 n.s.	
Three-chamber social interaction test	+/+ (+Vehicle)=16<br>+/+ (+CZP)=18<br>-/- (+Vehicle)=18<br>-/- (+CZP)=16	Time in close interaction (Empty-Empty)	+/+ (+Vehicle)	Empty (Left)	58.28	7.03	One-way ANOVA	0.8503	0.5479 n.s.	-	-	-	7a
				Empty (Right)	79.66	9.94							
			+/+ (+CZP)	Empty (Left)	80.42	11.14							
				Empty (Right)	84.44	12.15							
			-/- (+Vehicle)	Empty (Left)	75.72	6.44							
				Empty (Right)	88.50	11.12							
			-/- (+CZP)	Empty (Left)	75.44	11.97							
				Empty (Right)	91.75	15.18							
		Time in close interaction (Stranger-Empty)	+/+ (+Vehicle)	Stranger	212.47	11.99	One-way ANOVA	45.70	< 0.0001	Tukey's	Stranger vs. Empty	< 0.0001	
				Empty	45.91	4.85							
			+/+ (+CZP)	Stranger	190.69	13.32						< 0.0001	
				Empty	73.78	8.68							
			-/- (+Vehicle)	Stranger	199.58	12.01						< 0.0001	
				Empty	73.78	9.89							
			-/- (+CZP)	Stranger	169.50	11.72						< 0.0001	
				Empty	61.25	6.87							
		Time in close interaction (Familiar-Stranger)	+/+ (+Vehicle)	Familiar stranger	89.03	11.39	One-way ANOVA	8.766	< 0.0001	Tukey's	Familiar stranger vs. Novel stranger	0.0001	
				Novel stranger	177.03	13.49							
			+/+ (+CZP)	Familiar stranger	93.22	11.66						0.0018	
				Novel stranger	163.72	12.37							
			-/- (+Vehicle)	Familiar stranger	157.75	17.89						0.4889	
				Novel stranger	123.42	7.95							
			-/- (+CZP)	Familiar stranger	82.41	10.49						0.0095	
				Novel stranger	148.59	11.91							
		Preference index (control)	+/+ (+Vehicle)	Empty(Right) -Empty(Left)	6.78	4.29	One-way ANOVA	0.3510	0.7885 n.s.	-	-	-	7b
			+/+ (+CZP)		-0.20	5.79							
			-/- (+Vehicle)		0.73	5.30							
			-/- (+CZP)		3.27	5.23							
		Preference index (social preference)	+/+ (+Vehicle)	Stranger-Empty	31.94	1.98	One-way ANOVA	2.318	0.0839 n.s.	-	-	-	
			+/+ (+CZP)		22.28	3.28							
			-/- (+Vehicle)		23.53	2.95							
			-/- (+CZP)		24.31	2.72							
		Preference index (social novelty)	+/+ (+Vehicle)	Novel stranger -Familiar stranger	17.22	3.57	One-way ANOVA	9.100	< 0.0001	Tukey's	+/+(Veh) vs. -/-(Veh)	0.0001	
			+/+ (+CZP)		14.01	3.70					+/+(CZP) vs. -/-(Veh)	0.0010	
			-/- (+Vehicle)		-4.23	3.12					-/-(Veh) vs. -/-(CZP)	0.0007	
			-/- (+CZP)		15.03	2.82					other combinations	> 0.9050 n.s.	
		Time in chamber (Empty-Empty)	+/+ (+Vehicle)	Empty (Left)	191.06	15.02	One-way ANOVA	2.973	0.0011	Tukey's	Empty (Left) vs. Middle	> 0.9999 n.s.	
				Middle	173.59	14.05					Empty (Right) vs. Middle	0.6271 n.s.	
				Empty (Right)	235.34	16.26					Empty (Left) vs. Empty (Right)	0.9384 n.s.	
			+/+ (+CZP)	Empty (Left)	240.97	23.96					Empty (Left) vs. Middle	0.0052	
				Middle	130.06	17.85					Empty (Right) vs. Middle	0.0231	
				Empty (Right)	228.97	25.73					Empty (Left) vs. Empty (Right)	> 0.9999 n.s.	
			-/- (+Vehicle)	Empty (Left)	207.08	12.80					Empty (Left) vs. Middle	0.9827 n.s.	
				Middle	172.00	17.19					Empty (Right) vs. Middle	0.8372 n.s.	
				Empty (Right)	220.92	24.73					Empty (Left) vs. Empty (Right)	> 0.9999 n.s.	
			-/- (+CZP)	Empty (Left)	202.06	23.44					Empty (Left) vs. Middle	0.9805 n.s.	
				Middle	164.28	18.82					Empty (Right) vs. Middle	0.4429 n.s.	
				Empty (Right)	233.66	24.93					Empty (Left) vs. Empty (Right)	0.9955 n.s.	
		Time in chamber (Stranger-Empty)	+/+ (+Vehicle)	Stranger	350.94	10.71					Stranger vs. Middle	< 0.0001	
				Middle	100.50	7.65					Empty vs. Middle	0.2859 n.s.	
				Empty	148.56	8.06					Stranger vs. Empty	< 0.0001	

			+/+ (+CZP)	Stranger	315.22	17.86	One-way ANOVA	58.58	< 0.0001	Tukey's	Stranger vs. Middle	< 0.0001	7c
				Middle	104.92	9.86					Empty vs. Middle	0.0016	
				Empty	179.86	13.15					Stranger vs. Empty	< 0.0001	
			-/- (+Vehicle)	Stranger	324.33	9.78					Stranger vs. Middle	< 0.0001	
				Middle	91.81	6.21					Empty vs. Middle	< 0.0001	
				Empty	183.86	9.13					Stranger vs. Empty	< 0.0001	
			-/- (+CZP)	Stranger	308.06	16.51					Stranger vs. Middle	< 0.0001	
				Middle	128.16	19.01					Empty vs. Middle	0.7397 n.s.	
				Empty	163.78	16.79					Stranger vs. Empty	< 0.0001	
		Time in chamber (Familiar-Stranger)	+/+ (+Vehicle)	Familiar stranger	187.19	15.84	One-way ANOVA	49.81	< 0.0001	Tukey's	Familiar stranger vs. Middle	< 0.0001	
				Middle	87.84	7.51					Novel stranger vs. Middle	< 0.0001	
				Novel stranger	324.97	16.82					Familiar stranger vs. Novel stranger	< 0.0001	
			+/+ (+CZP)	Familiar stranger	211.03	15.34					Familiar stranger vs. Middle	< 0.0001	
				Middle	80.81	5.94					Novel stranger vs. Middle	< 0.0001	
				Novel stranger	308.17	15.26					Familiar stranger vs. Novel stranger	< 0.0001	
			-/- (+Vehicle)	Familiar stranger	283.97	16.08					Familiar stranger vs. Middle	< 0.0001	
				Middle	84.39	8.74					Novel stranger vs. Middle	< 0.0001	
				Novel stranger	231.64	10.56					Familiar stranger vs. Novel stranger	0.1385 n.s.	
			-/- (+CZP)	Familiar stranger	193.53	12.73					Familiar stranger vs. Middle	0.0017	
				Middle	112.50	12.14					Novel stranger vs. Middle	< 0.0001	
				Novel stranger	293.97	13.59					Familiar stranger vs. Novel stranger	< 0.0001	
		Preference index (control)	+/+ (+Vehicle)	Empty(Right) -Empty(Left)	5.11	3.08	One-way ANOVA	0.6055	0.6138 n.s.	-	-	-	7d
			+/+ (+CZP)		-2.40	5.27							
			-/- (+Vehicle)		-0.82	4.53							
			-/- (+CZP)		3.33	4.54							
		Preference index (social preference)	+/+ (+Vehicle)	Stranger-Empty	20.18	1.63	One-way ANOVA	1.478	0.2288 n.s.	-	-	-	
			+/+ (+CZP)		13.27	2.82							
			-/- (+Vehicle)		13.80	1.75							
			-/- (+CZP)		16.24	3.63							
		Preference index (social novelty)	+/+ (+Vehicle)	Novel stranger -Familiar stranger	13.39	3.06	One-way ANOVA	9.040	< 0.0001	Tukey's	+/+(Veh) vs. -/-(Veh)	< 0.0001	
			+/+ (+CZP)		9.38	2.83					+/+(CZP) vs. -/-(Veh)	0.0020	
			-/- (+Vehicle)		-4.59	2.48					-/-(Veh) vs. -/-(CZP)	0.0013	
			-/- (+CZP)		10.34	2.34					other combinations	> 0.7201 n.s.	
		Number of entries into each chamber	+/+ (+Vehicle)	Empty (Left)	6.63	0.50	One-way ANOVA	1.806	0.0914 n.s.	-	-	-	S7g
				Empty (Right)	7.00	0.61							
			+/+ (+CZP)	Empty (Left)	5.78	0.67							
				Empty (Right)	6.67	0.86							
			-/- (+Vehicle)	Empty (Left)	5.28	0.50							
				Empty (Right)	4.83	0.62							
			-/- (+CZP)	Empty (Left)	4.94	0.53							
				Empty (Right)	5.31	0.59							
			+/+ (+Vehicle)	Stranger	7.94	0.44	One-way ANOVA	1.153	0.3345 n.s.	-	-	-	
				Empty	6.63	0.45							
			+/+ (+CZP)	Stranger	9.06	0.85							
				Empty	8.89	0.96							
			-/- (+Vehicle)	Stranger	8.50	0.68							
				Empty	7.67	0.54							
			-/- (+CZP)	Stranger	8.88	0.86							
				Empty	7.75	1.04							
			+/+ (+Vehicle)	Familiar stranger	7.63	0.66	One-way ANOVA	1.185	0.3159 n.s.	-	-	-	
				Novel stranger	8.38	0.53							
			+/+ (+CZP)	Familiar stranger	8.78	0.85							
				Novel stranger	10.44	1.03							
			-/- (+Vehicle)	Familiar stranger	8.33	0.66							
				Novel stranger	8.28	0.66							
			-/- (+CZP)	Familiar stranger	8.38	0.76							
				Novel stranger	8.25	0.84							
			+/+ (+Vehicle)	Middle (Empty-Empty)	12.69	0.99	One-way ANOVA	2.358	0.0799 n.s.	-	-	-	
			+/+ (+CZP)		12.17	1.40							
			-/- (+Vehicle)		9.50	1.05							
			-/- (+CZP)		9.31	0.99							
			+/+ (+Vehicle)	Middle (Stranger-Empty)	13.88	0.83	One-way ANOVA	0.8743	0.4592 n.s.	-	-	-	
			+/+ (+CZP)		17.22	1.76							
			-/- (+Vehicle)		15.61	1.19							
			-/- (+CZP)		15.81	1.80							
			+/+ (+Vehicle)	Middle (Familiar-Stranger)	15.25	1.18	One-way ANOVA	1.301	0.2817 n.s.	-	-	-	
			+/+ (+CZP)		18.89	1.79							
			-/- (+Vehicle)		16.50	1.27							
			-/- (+CZP)		15.44	1.54							
		Distance moved	+/+ (+Vehicle)	Empty-Empty	26.82	0.88	One-way ANOVA	1.012	0.3932 n.s.	-	-	-	S7e
			+/+ (+CZP)		26.29	1.71							
			-/- (+Vehicle)		29.11	0.88							
			-/- (+CZP)		26.63	1.49							
			+/+ (+Vehicle)	Stranger-Empty	20.98	0.75	One-way ANOVA	1.829	0.1507 n.s.	-	-	-	
			+/+ (+CZP)		23.57	1.52							
			-/- (+Vehicle)		24.97	1.03							
			-/- (+CZP)		24.01	1.47							
			+/+ (+Vehicle)	Familiar-Stranger	20.02	0.85	One-way ANOVA	1.345	0.2678 n.s.	-	-	-	
			+/+ (+CZP)		23.21	1.35							
			-/- (+Vehicle)		22.69	1.12							
			-/- (+CZP)		21.89	1.34							
		Immobile time	+/+ (+Vehicle)	Empty-Empty	320.09	8.17	One-way ANOVA	1.326	0.2738 n.s.	-	-	-	S7f
			+/+ (+CZP)		338.53	14.56							
			-/- (+Vehicle)		313.72	6.87							
			-/- (+CZP)		338.78	13.18							
			+/+ (+Vehicle)	Stranger-Empty	393.19	6.78	One-way ANOVA	0.7833	0.5076 n.s.	-	-	-	
			+/+ (+CZP)		381.28	11.34							
			-/- (+Vehicle)		371.81	8.14							
			-/- (+CZP)		384.34	12.48							
			+/+ (+Vehicle)	Familiar-Stranger	407.88	7.61	One-way ANOVA	0.7564	0.5227 n.s.	-	-	-	
			+/+ (+CZP)		390.00	9.76							
			-/- (+Vehicle)		396.53	8.70							
			-/- (+CZP)		404.13	10.26							
Water T-maze test	+/+ (+Vehicle)=17<br>+/+ (+CZP)=17<br>-/- (+Vehicle)=17<br>-/- (+CZP)=17	Number of correct trials	+/+ (+Vehicle)	Acquisition trials-1	11.88	0.48					all combinations	> 0.9999 n.s.	
			+/+ (+CZP)		11.18	0.79							
			-/- (+Vehicle)		12.18	0.43							
			-/- (+CZP)		11.76	0.57							
			+/+ (+Vehicle)	Acquisition trials-2	13.76	0.39					all combinations	> 0.9999 n.s.	
			+/+ (+CZP)		13.41	0.62							
			-/- (+Vehicle)		13.88	0.22							
			-/- (+CZP)		14.29	0.25							

			+/+ (+Vehicle)	Acquisition trials-3	14.76	0.14	Two-way ANOVA	Genotype 51.05 Treatment 118.1 Interaction 12.00	Genotype < 0.0001 Treatment < 0.0001 Interaction < 0.0001	Bonferroni's	all combinations	> 0.9999 n.s.	7e
			+/+ (+CZP)		14.82	0.10							
			-/- (+Vehicle)		14.47	0.15							
			-/- (+CZP)		14.94	0.06							
			+/+ (+Vehicle)	Reversal learning-1	8.35	0.66					+/+ (+Vehicle) vs. -/- (+Vehicle)	< 0.0001	
			+/+ (+CZP)		8.59	0.50					+/+ (+CZP) vs. -/- (+Vehicle)	< 0.0001	
			-/- (+Vehicle)		2.00	0.51					-/- (+Vehicle) vs. -/- (+CZP)	< 0.0001	
			-/- (+CZP)		7.29	0.55					Other combinations	> 0.9999 n.s.	
			+/+ (+Vehicle)	Reversal learning-2	13.59	0.86					+/+ (+Vehicle) vs. -/- (+Vehicle)	< 0.0001	
			+/+ (+CZP)		14.47	0.15					+/+ (+CZP) vs. -/- (+Vehicle)	< 0.0001	
			-/- (+Vehicle)		6.35	0.90					-/- (+Vehicle) vs. -/- (+CZP)	< 0.0001	
			-/- (+CZP)		14.29	0.17					Other combinations	> 0.9999 n.s.	
			+/+ (+Vehicle)	Reversal learning-3	14.12	0.88					+/+ (+Vehicle) vs. -/- (+Vehicle)	< 0.0001	
			+/+ (+CZP)		14.71	0.14					+/+ (+CZP) vs. -/- (+Vehicle)	< 0.0001	
			-/- (+Vehicle)		9.41	1.05					-/- (+Vehicle) vs. -/- (+CZP)	< 0.0001	
			-/- (+CZP)		14.71	0.11					Other combinations	> 0.9999 n.s.	
		Trials required for 5 consecutive correct arm choices	+/+ (+Vehicle)	Acquisition trials-1	9.53	0.73	Two-way ANOVA#	Genotype 9.243 Treatment 30.00 Interaction 3.002	Genotype < 0.0001 Treatment < 0.0001 Interaction 0.0006	Bonferroni's	all combinations	> 0.9999 n.s.	S7h
			+/+ (+CZP)		9.00	0.71							
			-/- (+Vehicle)		8.94	0.74							
			-/- (+CZP)		9.24	0.72							
			+/+ (+Vehicle)	Acquisition trials-2	6.06	0.53					all combinations	> 0.9999 n.s.	
			+/+ (+CZP)		7.00	0.71							
			-/- (+Vehicle)		7.06	0.67							
			-/- (+CZP)		5.71	0.59							
			+/+ (+Vehicle)	Acquisition trials-3	5.53	0.36					all combinations	> 0.9999 n.s.	
			+/+ (+CZP)		5.24	0.24							
			-/- (+Vehicle)		5.65	0.28							
			-/- (+CZP)		5.06	0.06							
			+/+ (+Vehicle)	Reversal learning-1	11.60	0.47					all combinations	Not determined#	
			+/+ (+CZP)		11.56	0.42							
			-/- (+Vehicle)		-	-							
			-/- (+CZP)		12.62	0.48							
			+/+ (+Vehicle)	Reversal learning-2	6.00	0.44					+/+ (+Vehicle) vs. -/- (+Vehicle)	0.0531 n.s.	
			+/+ (+CZP)		5.82	0.35					+/+ (+CZP) vs. -/- (+Vehicle)	0.0381	
			-/- (+Vehicle)		14.00	0.00					-/- (+Vehicle) vs. -/- (+CZP)	0.0279	
			-/- (+CZP)		5.65	0.27					Other combinations	> 0.9999 n.s.	
			+/+ (+Vehicle)	Reversal learning-3	5.00	0.00					+/+ (+Vehicle) vs. -/- (+Vehicle)	0.0005	
			+/+ (+CZP)		5.47	0.41					+/+ (+CZP) vs. -/- (+Vehicle)	0.0042	
			-/- (+Vehicle)		9.22	0.78					-/- (+Vehicle) vs. -/- (+CZP)	0.0056	
			-/- (+CZP)		5.53	0.29					Other combinations	> 0.9999 n.s.	
		Latency to escape	+/+ (+Vehicle)	Acquisition trials-1	13.66	0.89	Two-way ANOVA	Genotype 55.74 Treatment 125.1 Interaction 8.246	Genotype < 0.0001 Treatment < 0.0001 Interaction < 0.0001	Bonferroni's	all combinations	> 0.9999 n.s.	7f
			+/+ (+CZP)		14.47	1.18							
			-/- (+Vehicle)		14.62	0.75							
			-/- (+CZP)		14.40	1.05							
			+/+ (+Vehicle)	Acquisition trials-2	8.22	0.52					all combinations	> 0.9999 n.s.	
			+/+ (+CZP)		8.62	0.55							
			-/- (+Vehicle)		9.31	0.43							
			-/- (+CZP)		8.89	0.46							
			+/+ (+Vehicle)	Acquisition trials-3	5.86	0.20					all combinations	> 0.9999 n.s.	
			+/+ (+CZP)		6.24	0.29							
			-/- (+Vehicle)		7.58	0.53							
			-/- (+CZP)		6.98	0.35							
			+/+ (+Vehicle)	Reversal learning-1	12.78	0.72					+/+ (+Vehicle) vs. -/- (+Vehicle)	< 0.0001	
			+/+ (+CZP)		14.54	0.54					+/+ (+CZP) vs. -/- (+Vehicle)	< 0.0001	
			-/- (+Vehicle)		22.91	1.33					-/- (+Vehicle) vs. -/- (+CZP)	< 0.0001	
			-/- (+CZP)		14.88	0.88					Other combinations	> 0.9999 n.s.	
			+/+ (+Vehicle)	Reversal learning-2	6.24	0.29					+/+ (+Vehicle) vs. -/- (+Vehicle)	< 0.0001	
			+/+ (+CZP)		6.31	0.19					+/+ (+CZP) vs. -/- (+Vehicle)	< 0.0001	
			-/- (+Vehicle)		15.66	1.44					-/- (+Vehicle) vs. -/- (+CZP)	< 0.0001	
			-/- (+CZP)		6.72	0.31					Other combinations	> 0.9999 n.s.	
			+/+ (+Vehicle)	Reversal learning-3	5.41	0.23					+/+ (+Vehicle) vs. -/- (+Vehicle)	< 0.0001	
			+/+ (+CZP)		5.62	0.15					+/+ (+CZP) vs. -/- (+Vehicle)	< 0.0001	
			-/- (+Vehicle)		11.03	1.16					-/- (+Vehicle) vs. -/- (+CZP)	< 0.0001	
			-/- (+CZP)		5.64	0.21					Other combinations	> 0.9999 n.s.	

Since no *PX-RICS*$^{-/-}$ (+Vehicle) mice could make a 5 consecutive correct arm choice in the reversal learning-1, the corresponding data were removed in the two-way ANOVA.
